# Homocysteine and *S*-adenosyl-*L*-homocysteine impair development and methylation in yeast and flies

**DOI:** 10.1242/dmm.052802

**Published:** 2026-06-01

**Authors:** Markus S. Brunner, Hansjörg Habisch, Jutta Hafner, Maximilian Mack, Zoriana Novosiadla, Andrea C. López Díaz, Heimo Wolinski, Gerald N. Rechberger, Tobias Eisenberg, Tobias Madl, Brigitte Pertschy, Ronald P. Kühnlein, Oksana Tehlivets

**Affiliations:** ^1^ Institute of Molecular Biosciences, University of Graz, 8010 Graz, Austria; ^2^ Otto Loewi Research Center, Medicinal Chemistry, Medical University of Graz, 8010 Graz, Austria; ^3^ Division of General Radiology, Department of Radiology, Medical University of Graz, 8036 Graz, Austria

**Keywords:** *S*-adenosyl-*L*-homocysteine, Homocysteine, Protein and phospholipid methylation, Yeast, *Drosophila*

## Abstract

*S*-adenosyl-*L*-homocysteine (SAH), the product inhibitor of *S*-adenosyl-*L*-methionine-dependent methyltransferases, and its degradation product homocysteine (Hcy) are evolutionarily conserved master regulators of methylation metabolism, which is mediated by more than 200 methyltransferases in humans. Hyperhomocysteinemia (HHcy), characterized by elevated levels of Hcy in the blood, is an independent risk factor for atherosclerosis, a strong predictor of cardiovascular mortality and can cause associated pathology by interfering with methylation-dependent processes. Here, we developed a *Drosophila melanogaster* fly dietary model of HHcy and a *Drosophila melanogaster* genetic SAH accumulation model and compared them to corresponding *Saccharomyces cerevisiae* yeast models to reveal evolutionarily conserved methylation pattern changes responsive to elevation of Hcy levels. Feeding *Drosophila* an Hcy-containing diet or growing yeast on Hcy-supplemented medium, similarly to genetically blocking SAH degradation, led to SAH accumulation, developmental delay and growth defects. Furthermore, dietary or genetically induced SAH accumulation caused impaired phospholipid and protein methylation in both model organisms. Identification and functional characterization of evolutionarily conserved SAH-dependent methylation targets responsive to elevation of Hcy and/or SAH levels will reveal mechanisms of SAH toxicity in HHcy and help to decipher their role in associated pathologies.

## Teaser

**Summary:** Developmental and methylation defects in yeast and fly models of hyperhomocysteinemia suggest that *S*-adenosyl-*L*-homocysteine accumulation mediates homocysteine-mediated cardiovascular disease in humans.

## INTRODUCTION

Cardiovascular disease (CVD), the leading cause of death worldwide, is still insufficiently understood (Lefkowitz and Willerson, 2001; Morita et al., 2005; Skeete and DiPette, 2017; Alani et al., 2014). Hyperhomocysteinemia (HHcy), characterized by elevation of homocysteine (Hcy) concentration in the blood, is an independent risk factor for the development of atherosclerosis, increases CVD risk in combination with hypercholesterolemia (Refsum et al., 1998), is associated with cardiac pathology (Burke et al., 2002; Fournier et al., 2015; Alter et al., 2010; Nasir et al., 2007; Jin et al., 2021) and is a strong predictor of CVD mortality (Vollset et al., 2001; Zylberstein et al., 2004; Zhang et al., 2020; Nygard et al., 1997).

Using a rabbit model, we recently showed that Hcy independently of cholesterol alters aortic wall structure and functionality as well as leads to deregulation of lipoprotein metabolism (Tehlivets et al., 2024). Elevation of plasma Hcy levels by intravenous injections of Hcy into rabbits fed a diet blocking Hcy degradation leads to impaired vascular reactivity of the aorta, accumulation of compromised, morphologically altered cells and deregulation of lipid metabolism in the aortic wall, as well as disorganization of aortic collagen and elastin (Tehlivets et al., 2024). Furthermore, elevation of Hcy levels in rabbits fed a diet blocking Hcy degradation was associated with a drop of total methylated arginine in proteins as well as decreased levels of creatine, which requires methylation for its synthesis (Tehlivets et al., 2024), suggesting involvement of deficient methylation in the pathological consequences associated with elevated Hcy.

Inhibition of methylation is an understudied mechanism of how elevated Hcy may lead to pathological consequences. There are more than 200 putative *S*-adenosyl-*L*-methionine (SAM)-dependent methyltransferases in humans (Petrossian and Clarke, 2011). As a side product of methylation, they release *S*-adenosyl-*L*-homocysteine (SAH), which is a universal product inhibitor of SAM-dependent methylation (Tehlivets et al., 2013). SAH has to be quickly degraded to Hcy and adenosine in a reaction catalyzed by SAH hydrolase (SAHH) in order to prevent SAH-mediated inhibition of SAM-dependent methylation (Tehlivets et al., 2013). An accumulation of Hcy, due to inability of its quick removal after SAH degradation, reverses the direction of the equilibrium of the SAH hydrolytic reaction potentiating SAH buildup (Tehlivets et al., 2013). In accordance, both elevated SAH and Hcy levels are known to inhibit methylation (Esse et al., 2019).

Methylation is an important regulatory mechanism affecting various macromolecules and a wide variety of cellular processes (Tehlivets et al., 2013), and its dysregulation is increasingly recognized as a risk factor for CVD development (Muka et al., 2016; Haas et al., 2013; Agha et al., 2019; Zhou et al., 2021). CVD has been linked to increased levels of SAH (Kerins et al., 2001). Lowering of plasma Hcy by vitamin supplementation does not reduce HHcy-associated cardiovascular events (Loscalzo, 2006; Lonn et al., 2006; Bonaa et al., 2006), likely because it does not decrease plasma SAH levels (Green et al., 2010), suggesting a central role of SAH in Hcy-associated pathology.

Here, we show that Hcy supplementation, similarly to genetic impairment of SAHH expression, leads to SAH accumulation, developmental delay, growth defects, and impaired protein and phospholipid (PL) methylation in yeast and in *Drosophila*. Although Hcy and/or SAH accumulation led to a decreased total protein asymmetrically dimethylated arginine to arginine (ADMA/Arg) ratio along with impaired histone and PL methylation in yeast, total protein ADMA/Arg was unaffected in *Drosophila*, whereas histone and PL methylation were sensitive to Hcy and/or SAH accumulation in fly models. Furthermore, *Drosophila* larvae subject to knockdown of the SAHH-encoding gene showed delayed development, reduced size and poor viability.

## RESULTS

### Differential response of SAH and SAM to Hcy supplementation or genetic SAH hydrolase inhibition in yeast and *Drosophila*

**Fig. 1.**
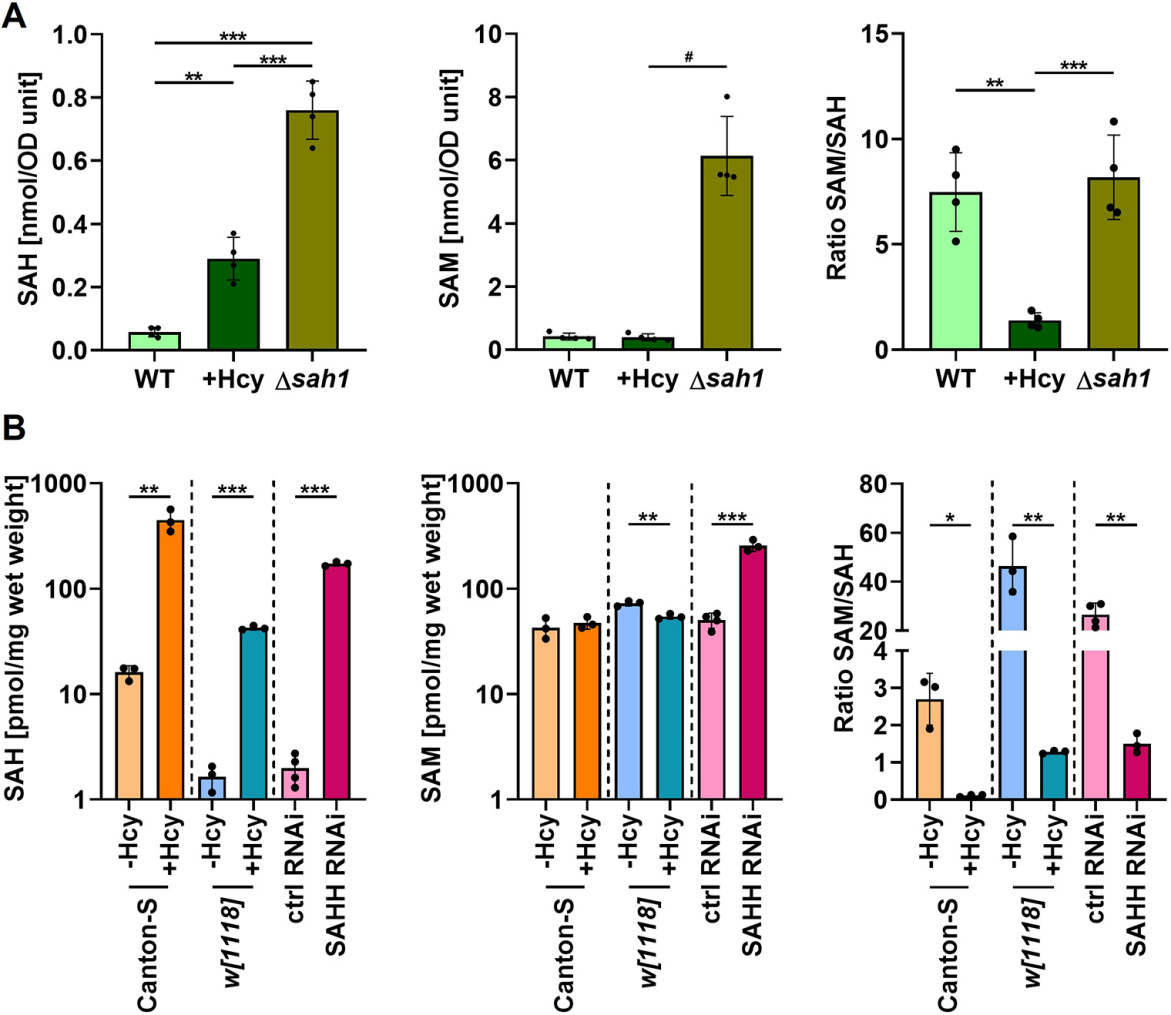
***S*-adenosyl-*L*-homocysteine (SAH) and *S*-adenosyl-*L*-methionine (SAM) accumulation in yeast and *Drosophila* dietary and genetic models.** (A) Total SAH and SAM levels as well as SAM/SAH ratios in wild-type yeast grown in the presence or absence of 5 mM homocysteine (Hcy), and in the yeast Δ*sah1* mutant grown without Hcy supplementation (*n*=4). OD, optical density. Data were collected in two independent analyses, each with two biological replicates. (B) Total SAH and SAM levels as well as SAM/SAH ratios in migratory L3 Canton-S and *w[1118] Drosophila* larvae grown on standard fly food or food containing 20 mM Hcy, as well as in *Drosophila* larvae ubiquitously expressing an mCherry RNA interference (RNAi) transgene (control) or a SAH hydrolase (SAHH) RNAi transgene grown on standard food (*n*=3-4). Data were collected independently for both fly lines of the dietary model as well as for genetic model, with three to four replicates, each using five larvae. For A, statistics were calculated with unpaired two-tailed Student’s *t*-test or Mann–Whitney *U*-test pairwise comparisons with Bonferroni correction for multiple testing. For B, statistics were calculated with unpaired two-tailed Student’s *t*-tests (parametric, *P≤0.05, **P≤0.01, ***P≤0.001; non-parametric, ^#^*P*≤0.05). All statistics are shown in Dataset 3.

We have shown previously that Hcy supplementation leads to SAH accumulation in wild-type yeast cells and that deletion of the gene encoding SAHH (Δ*sah1*) results in SAH accumulation independently of Hcy supplementation (Visram et al., 2018). Given that SAH is considered a competitive inhibitor of SAM-dependent methyltransferases, we aimed to directly compare SAH and SAM levels following Hcy supplementation and in the SAHH-deficient mutant. Consistent with our previous results, SAH accumulated in wild-type yeast supplemented with 5 mM Hcy (referred to as the yeast Hcy supplementation model hereafter) and the yeast Δ*sah1* mutant (termed the yeast SAHH genetic model hereafter), leading to a 5-fold and 13-fold increase compared to that in non-supplemented wild-type yeast, respectively (Fig. 1A, left). Whereas SAM levels were not increased in Hcy-supplemented wild-type yeast, they were significantly elevated in the yeast Δ*sah1* mutant in comparison to those in non-supplemented wild type (Fig. 1A, middle). This resulted in a significantly decreased SAM/SAH ratio in Hcy-supplemented wild-type yeast, but a largely unaltered SAM/SAH ratio in the yeast Δ*sah1* mutant (Fig. 1A, right). In line with the yeast results, *Drosophila* Canton-S wild-type larvae grown on food containing 20 mM Hcy (referred to as the fly Hcy supplementation model hereafter) showed more than a 25-fold increase in SAH levels compared to those in larvae grown on non-supplemented food (Fig. 1B, left). To confirm that SAH accumulation in our fly Hcy supplementation model is a universal response of *Drosophila* larvae to dietary Hcy supplementation, we subjected the genetically unrelated *w[1118]* strain to the same food regimen. Although the basal levels of SAH were lower in this genetic background, we found a similar 25-fold increase in SAH upon Hcy supplementation compared to that in the same genotype on regular food (Fig. 1B, left). To exclude the possibility that SAH accumulation is an indirect effect of dietary supplementation due to an orexigenic effect of Hcy, we measured larval food intake and found no significant difference between larvae on food±Hcy in either Canton-S or *w[1118]* strains (Fig. S1). Furthermore, consistent with the corresponding yeast model, Hcy supplementation did not increase SAM levels in *w[1118]* or Canton-S larvae; SAM levels were even slightly decreased in *w[1118]* larvae (Fig. 1B, middle). As a consequence of the strong SAH increase without a corresponding rise in SAM, the SAM/SAH ratios were strongly decreased in both fly backgrounds (Fig. 1B, right). Similar to those with Hcy supplementation, *Drosophila* larvae subjected to ubiquitous SAHH-encoding gene knockdown mediated by an *in vivo* RNA interference (RNAi) construct (referred to as the fly SAHH genetic model hereafter) showed a dramatic 85-fold increase in SAH levels compared to control larvae expressing an unrelated RNAi construct (mCherry RNAi) (Fig. 1B, left). The efficiency of SAHH knockdown in this model was over 70% (Fig. S2). In accordance with the yeast SAHH genetic model, SAM levels in SAHH knockdown larvae were significantly increased compared to those in control RNAi larvae (Fig. 1B, middle). Nevertheless, as SAH was increased more than SAM in this model, the SAM/SAH ratio remained decreased, albeit to a lesser extent than in the *Drosophila* dietary Hcy models (Fig. 1B, right). Collectively, the SAH accumulation response to Hcy supplementation or SAHH inhibition is very similar in yeast and *Drosophila*. Moreover, in both models, SAHH inhibition, but not Hcy treatment, additionally results in SAM accumulation. These findings prompted us to further investigate whether the differences between dietary and genetic models regarding SAM accumulation also correlate with distinct effects on growth and development.

### Growth defects and developmental delay in yeast and *Drosophila* models of SAH accumulation

**Fig. 2.**
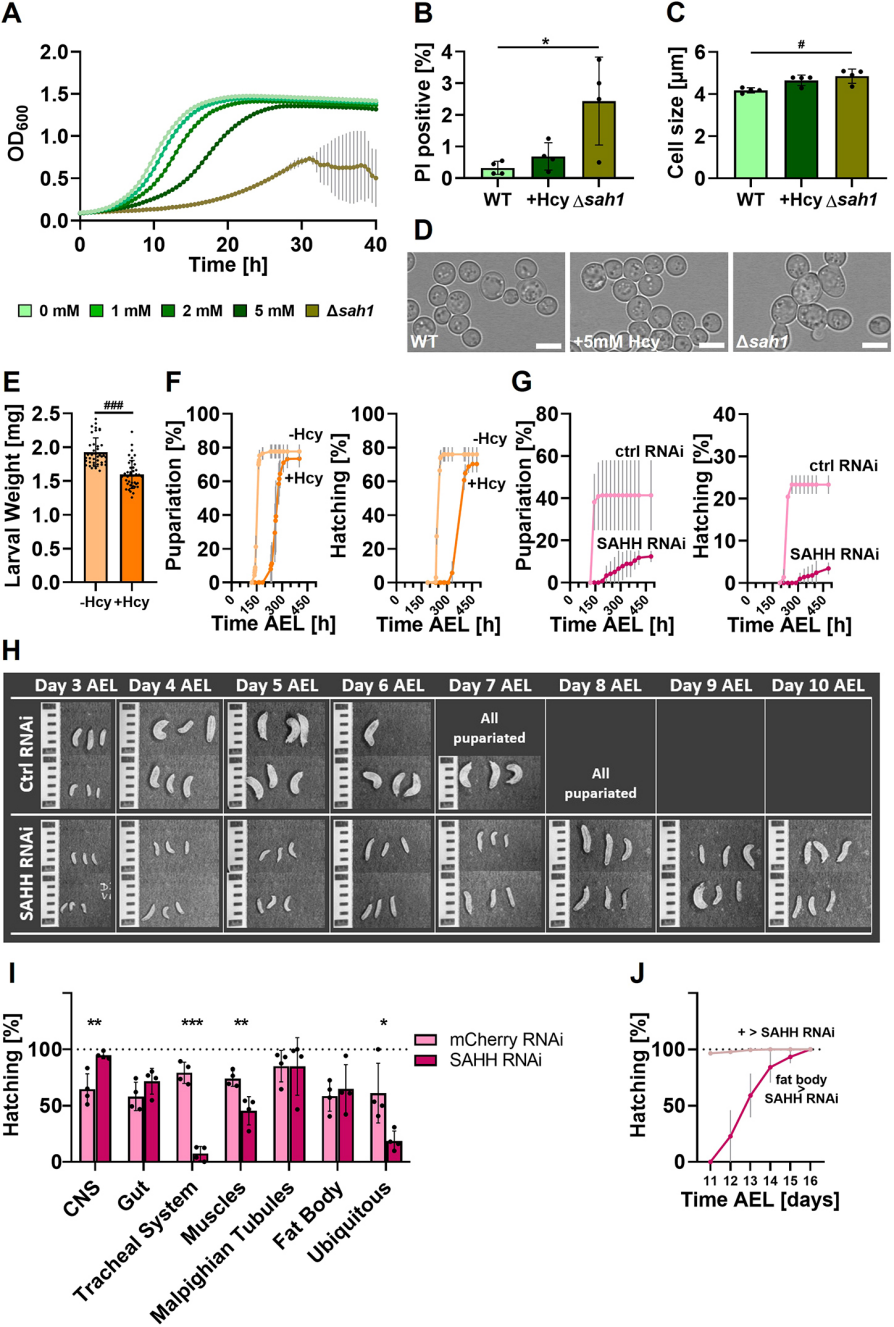
**Developmental delay and growth defects in yeast and *Drosophila* dietary and genetic models.** (A) Growth curves of wild-type yeast grown in the presence or absence of Hcy supplementation as indicated, and of the yeast Δ*sah1* mutant grown without Hcy supplementation measured over 40 h at optical density at 600 nm wavelength (OD_600_; *n*=2). (B) Cell-death analysis of wild-type yeast grown in the presence or absence of 5 mM Hcy, and of the yeast Δ*sah1* mutant grown without Hcy supplementation, analyzed after 30 h incubation. Cells were stained with propidium iodide (PI), with PI-positive cells being interpreted as dead cells (*n*=4). (C) Cell size analysis of wild-type yeast grown in the presence or absence of 5 mM Hcy, and of the yeast Δ*sah1* mutant grown without Hcy supplementation, analyzed after 30 h incubation (*n*=4). (D) Live-cell microscopy of wild-type yeast grown in the presence or absence of 5 mM Hcy, and of the yeast Δ*sah1* mutant grown without Hcy supplementation. Cells were analyzed after 40 h of cultivation. Scale bars: 5 µm. (E) Migratory L3 larval weight of Canton-S *Drosophila* larvae grown on standard fly food or food containing 20 mM Hcy (*n*=40). (F) Pupariation and adult hatching rates of Canton-S *Drosophila* larvae grown on standard fly food or food containing 20 mM Hcy (*n*=4). (G) Pupariation and adult hatching rates of *Drosophila* larvae ubiquitously expressing a mCherry RNAi transgene (control) or a SAHH RNAi transgene grown on standard food (*n*=2). (H) Size comparison of *Drosophila* larvae ubiquitously expressing a mCherry RNAi transgene (control) or a SAHH RNAi transgene grown on standard food. Photographs were taken daily, between the third and tenth day after egg laying (AEL). Scale guides in the photographs are given in 1 mm steps. (I) Hatching rates of *Drosophila* flies with organ-specific expression of mCherry RNAi (control) or SAHH RNAi grown on standard food (*n*=4). (J) Hatching rates of *Drosophila* progeny with fat body-targeted or uninduced SAHH knockdown (control) grown on standard food. Of note, selective counting started on day 11 AEL, and given values represent number of flies hatched relative to total hatched flies (*n*=3). For B, C, E and I, statistics were calculated with unpaired two-tailed Student’s *t*-test or Mann–Whitney *U*-test pairwise comparisons (parametric, *P≤0.05, **P≤0.01, ***P≤0.001; non-parametric, ^#^*P*≤0.05, ^###^*P*≤0.001). All statistics are shown in Dataset 3.

Tight regulation of SAH degradation is essential, as evidenced by the embryonic lethality of homozygous SAH hydrolase mutant mice (Miller et al., 1994). Similarly, the yeast SAHH genetic model (Δ*sah1*) exhibits a massive growth defect (Malanovic et al., 2008; Fig. 2A), which cannot be rescued by methionine supplementation (Visram et al., 2018). This suggests that SAH accumulation is causal of the growth defect triggered by the block in SAH hydrolysis. We hypothesized that SAH accumulation driven by Hcy supplementation would similarly impair growth in yeast. To test this, we measured growth of wild-type yeast in the absence (0 mM) or presence of increasing Hcy concentrations (1 mM, 2 mM or 5 mM) in the medium. Indeed, we observed dose-dependent growth inhibition with increasing Hcy concentration. An even stronger growth defect was observed in the yeast SAHH genetic model grown without Hcy (Fig. 2A). Although cultures of the yeast SAHH genetic model never reach stationary phase density, the Hcy-supplemented yeast did under all tested Hcy concentrations (Fig. 2A). In accordance, cultures of the yeast SAHH genetic model exhibited significantly higher number of propidium iodide (PI)-stained cells, indicating increased cell death, whereas Hcy-supplemented wild-type yeast cells showed survival rates comparable to those of untreated cells (Fig. 2B). Furthermore, cells of the yeast SAHH genetic model were significantly larger than wild-type yeast cells, in accordance with altered light scattering (Fig. 2C; Fig. S3A,C). In addition, aggregation of the yeast Δ*sah1* mutant cells was observed (Fig. 2D), in line with our previous observations of multi-budding and altered morphology of yeast Δ*sah1* mutant cells (Tehlivets et al., 2004). In accordance, the yeast Hcy supplementation model also exhibited a non-significant trend to increased cell size (Fig. 2C), while cell morphology and light scattering were unchanged from that in non-supplemented wild-type yeast (Fig. 2D; Fig. S3A,B).

To compare the developmental progress and success of the fly Hcy supplementation model to controls on regular food, we assessed the following parameters: the wet weight of individuals at migratory L3 larval stage; and the time of/survival rate at two developmental transition states – pupariation (larval to pupal) and hatching (pupal to adult). Migratory L3 larvae from the Canton-S fly Hcy supplementation model, similarly to *w[1118]*, grown on Hcy-containing food showed significantly lower body weight compared to that of respective larvae grown on food without Hcy supplementation (Fig. 2E; Fig. S4A). Moreover, pupariation and hatching were substantially delayed in the fly Hcy supplementation model compared to controls. The first pupae on Hcy food emerged 232 h after egg laying (AEL), compared to 136 h AEL on standard food (Fig. 2F, left). Additionally, the last larvae pupariated 401.5 h AEL on Hcy-containing food, compared to 232 h AEL on standard food (Fig. 2F, left). This extended pupariation phase in the population suggests an Hcy-dependent ontogenetic desynchronization by individually different growth retardation. Both aspects, developmental delay and extended developmental phase, are equally manifested when comparing Hcy-fed flies to controls at pupal hatching to adults (Fig. 2F, right). This indicates that Hcy exposure during larval feeding does not affect metamorphosis. Of note, despite the developmental delay, Hcy supplementation did not significantly decrease overall survival rates at pupariation or hatching in the fly Hcy supplementation model (Fig. 2F). Reduced larval body weight and developmental delay, combined with largely unaffected pupariation and hatching rates, appears to be a universal signature of Hcy-fed flies. In support of this, the genotypically unrelated *w[1118]* stock displayed a developmental phenotype similar to that of Canton-S in the fly Hcy supplementation model (compare Fig. S4 to Fig. S2E,F)

In accordance with SAH accumulation playing a central role in growth and development, larvae of the fly SAHH genetic model were also retarded at pupariation and adult hatching (Fig. 2G); however, the defect was much stronger than in the Hcy supplementation model. The first pupae of the genetic SAHH model developed 192 h AEL, compared to 144 h AEL in the case of control larvae with normal SAHH activity (Fig. 2G). Although the onset of the developmental delay was similar in the fly Hcy supplementation and SAHH genetic models, the pupariation phase at the population level was much more extended in the genetic model (compare Fig. 2F to G). Consistently, not only was the growth of larvae subjected to ubiquitous SAHH-encoding gene knockdown retarded, but size heterogeneity of larvae of the same chronological age (Fig. 2H) also indicated ontogenetic desynchronization. In line with the severe growth phenotype, but unlike the Hcy supplementation model, drastically reduced survival rates by 70% and 85% at pupariation and adult hatching, respectively, characterize the SAHH genetic model (Fig. 2G). These data suggest that chronic growth retardation in response to global reduction in SAHH activity eventually results in developmental arrest and death during larval development for the majority of individuals.

To address the organ-specific relevance of SAHH activity for proper development, we targeted knockdown of the SAHH-encoding gene selectively to different organs and scored for the adult hatching rate of the respective flies compared to that in controls subject to organ-specific expression of an mCherry RNAi control construct (Fig. 2I). SAHH-encoding gene knockdown in the tracheal system (insect respiratory system) and in muscle significantly reduced the hatching rate, as did the ubiquitous gene knockdown as shown above (Fig. 2I). In contrast, targeted knockdown of the SAHH-encoding gene in the central nervous system (CNS) neurons, the endocrine cells of the gut or the Malpighian tubules (insect kidneys) did not impact hatching (Fig. 2I). Of note, the organ specificity of SAHH knockdown at L3 larval stage was confirmed by GFP-reporter gene control crosses (Fig. S5). Interestingly, targeted SAHH impairment in the fat body (insect liver and adipose tissue equivalent) did not significantly affect developmental success, but caused developmental delay and ontogenetic phase extension at the population level (Fig. 2J), reminiscent of the fly Hcy supplementation model. Collectively, the fly Hcy supplementation and SAHH genetic models revealed that SAH accumulation affects *Drosophila* growth and development. Characterization of the lethal phase and the disclosure of the mechanisms responsible for phenotype severity of the SAHH genetic model, as well as developmental delay of the dietary model, deserve future research attention. We also present first evidence of organ-selective roles of SAHH in these processes. It is noteworthy that melanotic tumors were frequently observed in larvae subject to ubiquitous SAHH-encoding gene knockdown (Fig. S6), which might contribute to death during development. Given the central role of SAH as inhibitor of SAM-dependent methyltransferases, we asked next whether the methylation profiles of PLs and proteins were altered in the yeast and fly models.

### Hcy supplementation, similarly to genetic SAH hydrolase inhibition, leads to impaired PL methylation in yeast and *Drosophila*

**Fig. 3.**
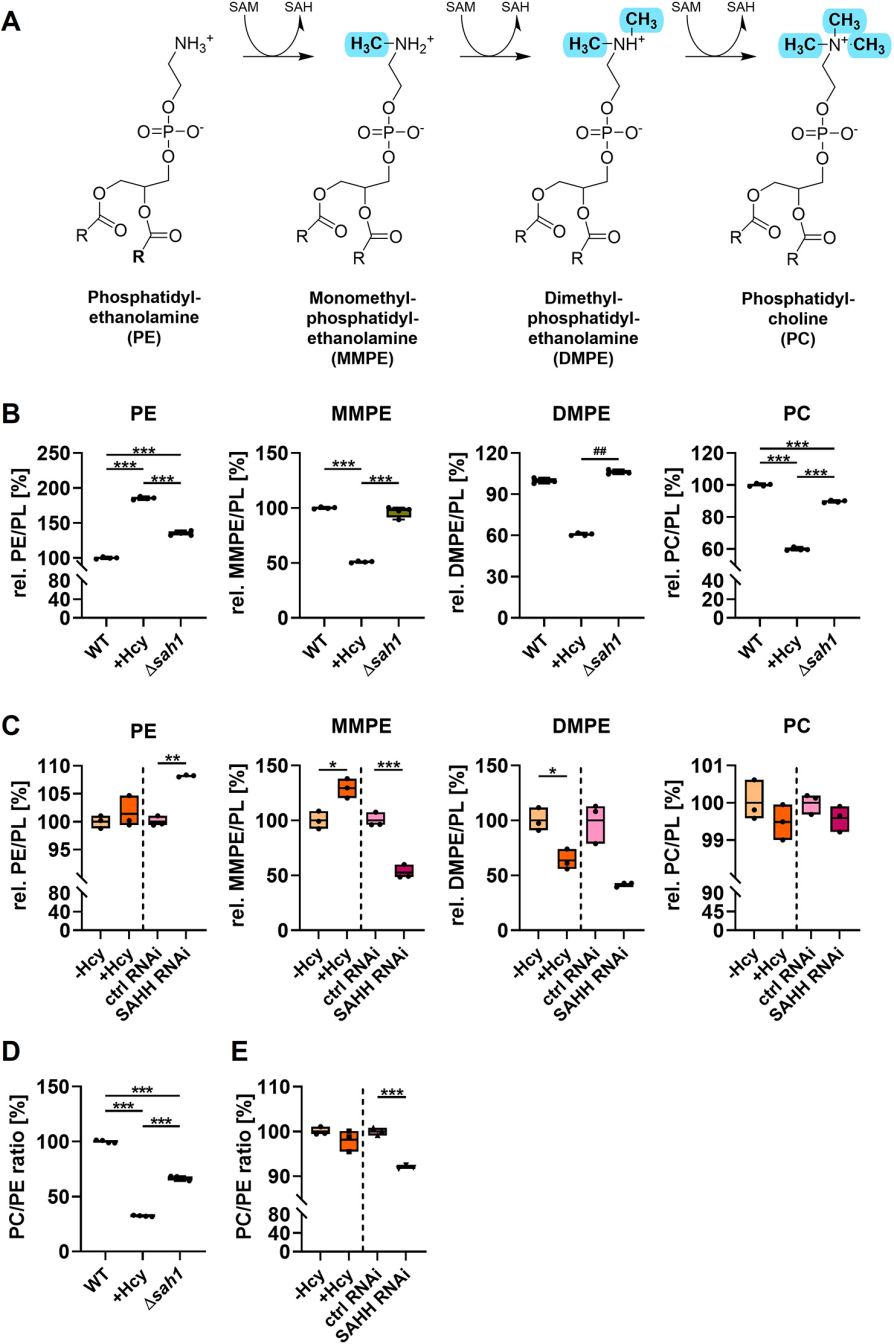
**Relative phospholipid (PL) contents in yeast and *Drosophila* dietary and genetic models.** (A) Schematic depiction of *S*-adenosyl-*L*-methionine (SAM)-dependent three-step methylation from phosphatidylethanolamine (PE) to phosphatidylcholine (PC). Schematic was prepared in ChemDraw (Version 23.1.1). (B) PE, monomethylphosphatidylethanolamine (MMPE), dimethylphosphatidylethanolamine (DMPE) and PC levels in wild-type yeast grown in the presence or absence of 5 mM Hcy, and in the yeast Δ*sah1* mutant grown without Hcy supplementation, relative to total analyzed PLs (*n*=4). Box plots represent peak integrals normalized to internal standard peak integrals relative to total analyzed PLs and normalized to the respective control group. (C) PE, MMPE, DMPE and PC levels in migratory L3 Canton-S *Drosophila* larvae grown on standard fly food or food containing 20 mM Hcy, and in L3 *Drosophila* larvae ubiquitously expressing a mCherry RNAi transgene (control) or a SAHH RNAi transgene grown on standard food, relative to total analyzed PLs (*n*=3). Box plots represent peak integrals normalized to internal standard peak integrals relative to total analyzed PLs and normalized to the respective control group. (D) PC/PE ratios in wild-type yeast grown in the presence of 5 mM Hcy, and in the yeast Δ*sah1* mutant grown without Hcy supplementation, relative to non-supplemented wild-type yeast PC/PE ratios. Box plot represents ratios of peak integrals normalized to internal standard peak integrals (*n*=4). (E) PC/PE ratios in migratory L3 Canton-S *Drosophila* larvae grown food containing 20 mM Hcy relative to larvae grown on standard food, and in L3 *Drosophila* larvae ubiquitously expressing a mCherry RNAi transgene (control) relative to larvae expressing a SAHH RNAi transgene, both grown on standard food. Box plot represents ratios of peak integrals normalized to internal standard peak integrals (*n*=3). For B and D, statistics were calculated with unpaired two-tailed Student’s *t*-test or Mann–Whitney *U*-test pairwise comparisons with Bonferroni or Games-Howell correction for multiple testing. For C and E, statistics were calculated with unpaired two-tailed Student’s *t*-test or Mann–Whitney *U*-test pairwise comparisons (parametric, *P≤0.05, **P≤0.01, ***P≤0.001; non-parametric, ^##^*P*≤0.01). All statistics are shown in Dataset 3.

PL methylation is a major consumer of SAM in both yeast and mammals (Ye et al., 2017; Noga et al., 2003), as the synthesis of phosphatidylcholine (PC) from phosphatidylethanolamine (PE) requires three sequential methylation steps via the intermediates monomethylphosphatidylethanolamine (MMPE) and dimethylphosphatidylethanolamine (DMPE) (Fig. 3A). Notably, PC can also be synthesized in an alternative pathway from choline (Malanovic et al., 2008). We have shown previously that PL methylation is sensitive to SAH accumulation as well as to Hcy supplementation in choline-free medium (Visram et al., 2018; Malanovic et al., 2008). Here, we investigated whether Hcy supplementation also impairs PC synthesis via the methylation pathway in the presence of choline. For this, we analyzed the levels of PE, MMPE, DMPE and PC in wild-type yeast grown in the presence or absence of Hcy in the medium and in the yeast Δ*sah1* mutant, as well as in larvae of the corresponding *Drosophila* Hcy supplementation and SAHH genetic models.

Indeed, in yeast, both Hcy-supplemented wild-type cells and the Δ*sah1* mutant exhibited significantly increased PE and significantly decreased PC levels, compared to those in non-supplemented wild-type yeast. Both the increase in PE and the decrease in PC were more pronounced in Hcy-supplemented wild type compared to the Δ*sah1* mutant (Fig. 3B, leftmost and rightmost graphs). In accordance, PC/PE ratios were significantly decreased in the Δ*sah1* mutant and even more strongly reduced in the Hcy-supplemented wild type (Fig. 3D). Furthermore, Hcy-supplemented wild type displayed decreased levels of the PL methylation intermediates MMPE and DMPE compared to untreated wild type. In contrast, the Δ*sah1* mutant exhibited MMPE and DMPE levels similar to those in the untreated wild type, which were, however, significantly higher than those in Hcy-supplemented cells (Fig. 3B, middle two graphs). Similarly, non-migratory L3 larvae of the *Drosophila* SAHH genetic model exhibited significantly increased PE levels, as well as tendentially decreased PC and DMPE levels, and significantly decreased MMPE levels, compared to migratory L3 control larvae (Fig. 3C). This resulted in a significantly decreased PC/PE ratio in response to SAHH downregulation (Fig. 3E). In contrast, larvae of the fly Hcy supplementation model exhibited a non-significant increase in PE, significantly increased MMPE levels, significantly decreased DMPE levels and tendentially decreased PC levels, compared to larvae grown in the absence of Hcy (Fig. 3C). Overall, this resulted in a weak trend to a decreased PC/PE ratio compared to that in non-supplemented larvae (Fig. 3E). We conclude that, although the magnitude of perturbations differs between model organisms and experimental conditions, both Hcy supplementation and SAHH deletion or depletion inhibit PL methylation, leading to alterations in the PC/PE ratio in yeast and *Drosophila*, supporting the existence of PL methylation in *Drosophila*.

### Hcy supplementation, similarly to genetic SAH hydrolase inhibition, leads to impaired protein methylation in yeast and *Drosophila*

**Fig. 4.**
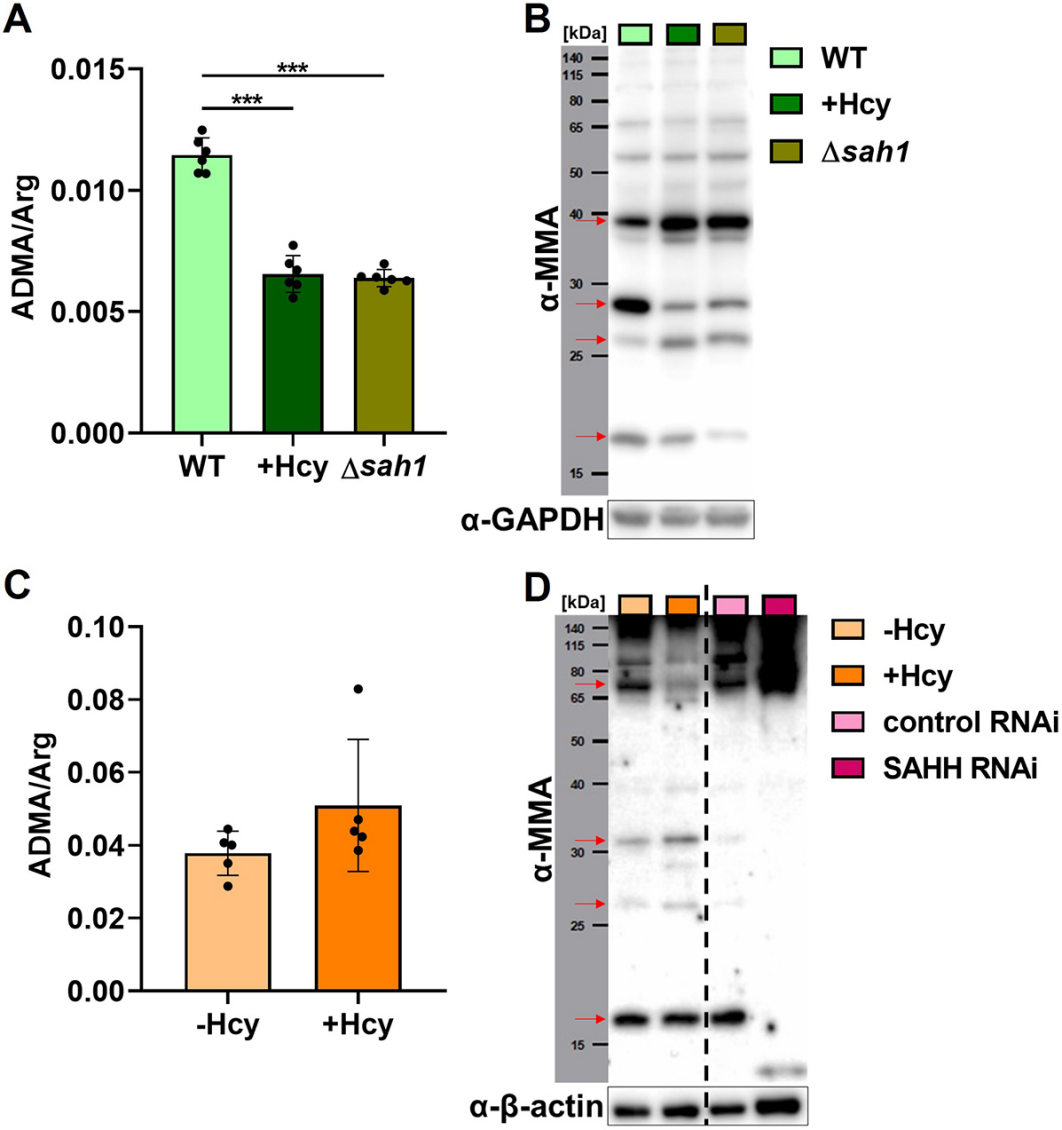
**Total protein asymmetrically dimethylated arginine to total arginine (ADMA/Arg) ratio in yeast and *Drosophila* dietary and genetic models.** (A) Nuclear magnetic resonance (NMR) analysis of ADMA/Arg ratios in wild-type yeast grown in the presence or absence of 5 mM Hcy supplementation, and in the yeast Δ*sah1* mutant grown without Hcy supplementation (*n*=6). (B) Western blot analysis of monomethylarginine (MMA)-containing proteins in wild-type yeast grown in the presence or absence of 5 mM Hcy and the yeast Δ*sah1* mutant grown without Hcy supplementation. Red arrows mark heights of distinct, unknown protein bands exhibiting altered intensities. (C) NMR analysis of total protein ADMA/Arg ratios in migratory L3 Canton-S *Drosophila* larvae grown on standard fly food or food containing 20 mM Hcy (*n*=5). (D) Western blot analysis of MMA-containing proteins in migratory L3 Canton-S *Drosophila* larvae grown on standard fly food or food containing 20 mM Hcy, and in L3 *Drosophila* larvae ubiquitously expressing a mCherry RNAi transgene (control) or a SAHH RNAi transgene grown on standard food. The dashed line acts as visual key separating the dietary and genetic models. Red arrows mark heights of distinct, unknown protein bands exhibiting altered intensities in the dietary and/or genetic model. For A, statistics were calculated with unpaired two-tailed Student’s *t*-tests with Bonferroni correction for multiple testing. For C, statistics were calculated with Mann–Whitney *U*-test pairwise comparisons (parametric, ***P≤0.001). All statistics are shown in Dataset 3.

Elevated SAH and Hcy levels are known to inhibit protein methylation (Esse et al., 2019). To analyze whether SAH accumulation due to Hcy supplementation or SAHH deficiency resulted in inhibition of protein methylation in our models, we first analyzed the total protein ADMA/Arg ratio in wild-type yeast grown in the presence of Hcy and in the yeast Δ*sah1* mutant. Indeed, wild-type yeast cultivated in the presence of Hcy, similarly to the Δ*sah1* mutant, exhibited decreased levels of ADMA relative to total Arg in proteins (Fig. 4A). Cultivation of wild-type yeast in the presence of Hcy also resulted in altered intensities of distinct protein bands of unknown identity detected by an anti-monomethylarginine (MMA)-specific antibody (Fig. 4B). Although the signals of two protein bands detected by the anti-MMA antibody were increased, two others were decreased (Fig. 4B). In contrast, the total protein ADMA/Arg ratio in the fly Hcy supplementation model was not significantly changed in comparison to that in non-supplemented larvae (Fig. 4C). In the fly Hcy supplementation and SAHH genetic models, the anti-MMA antibody detected differentially monomethylated proteins, similarly to observations made in yeast (compare Fig. 4B to D). These observations suggest that the inhibition of protein methylation by SAH may vary depending on specific proteins or residues involved and prompted us to also investigate methylation of protein lysines. To directly assess whether different methylation sites are differentially affected, we used site-specific antibodies to detect distinct methylated residues on defined proteins.

**Fig. 5.**
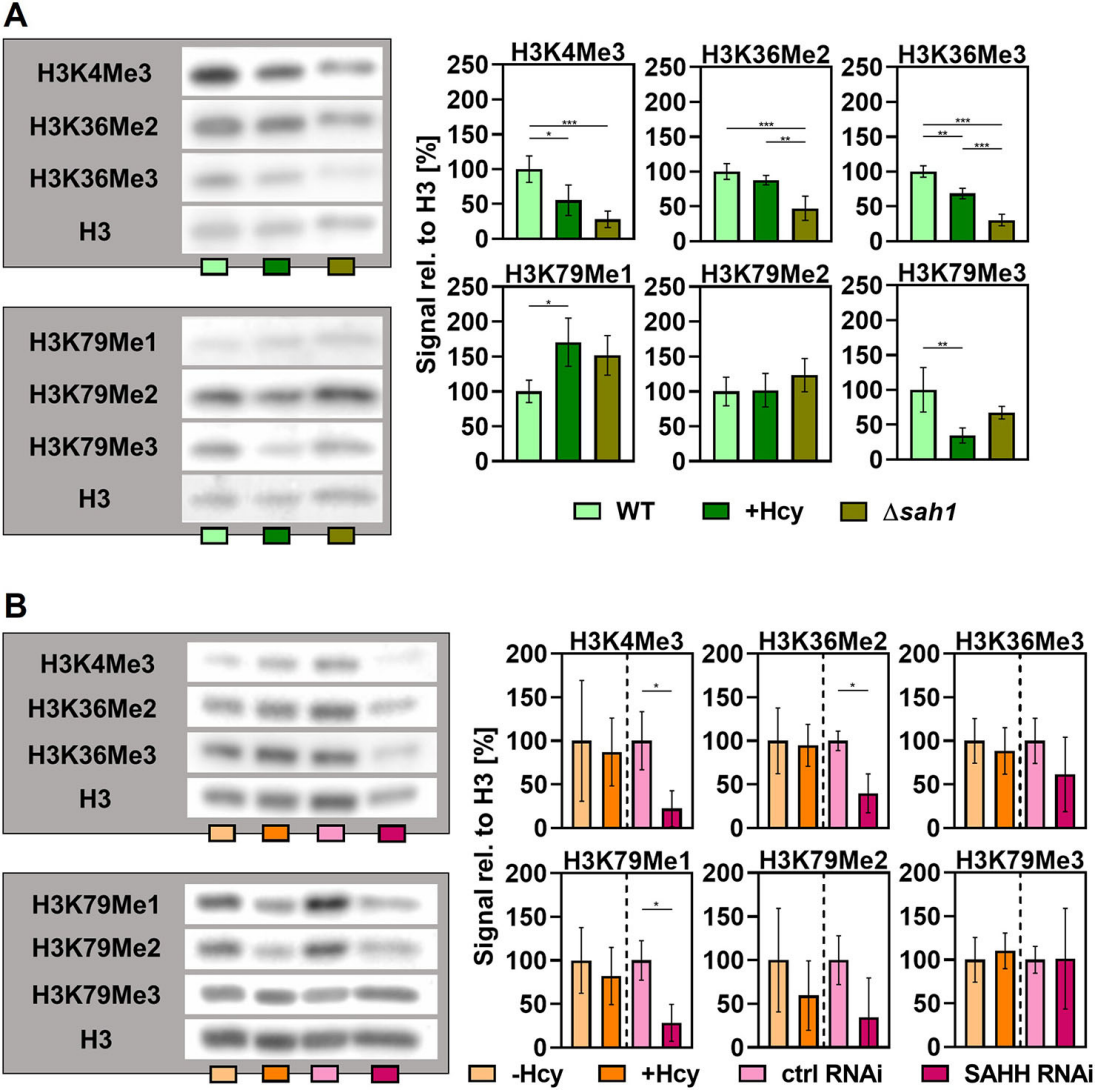
**Histone 3 (H3) lysine methylation in yeast and *Drosophila* dietary and genetic models.** (A) Western blot analyses of trimethylated H3K4, dimethylated and trimethylated H3K36, and monomethylated, dimethylated and trimethylated H3K79 in wild-type yeast grown in the presence or absence of 5 mM Hcy, and in the yeast Δ*sah1* mutant grown without Hcy supplementation. Bar charts represent quantified signals normalized to total H3 (*n*=4). (B) Western blot analyses of trimethylated H3K4, dimethylated and trimethylated H3K36, and monomethylated, dimethylated and trimethylated H3K79 in migratory L3 Canton-S *Drosophila* larvae grown on standard fly food or food containing 20 mM Hcy, and in L3 *Drosophila* larvae ubiquitously expressing a mCherry RNAi transgene (control) or a SAHH RNAi transgene grown on standard food. Bar charts represent quantified signals normalized to total H3 (*n*=3). For A, statistics were calculated with unpaired two-tailed Student’s *t*-test or Mann–Whitney *U*-test pairwise comparisons with Bonferroni correction for multiple testing. For B, statistics were calculated with unpaired two-tailed Student’s *t*-tests (parametric, *P≤0.05, **P≤0.01, ***P≤0.001). All statistics are shown in Dataset 3.

For this, we tested for potential inhibition of lysine methylation of histone 3 (H3) in response to Hcy supplementation. Indeed, wild-type yeast grown in the presence of Hcy exhibited significantly decreased levels of all analyzed tri-methylated sites – H3K4Me3, H3K36Me3 and H3K79Me3 – with H3K79Me3 being the one most affected (Fig. 5A). Similarly, the yeast Δ*sah1* mutant showed a significant decrease in tri-methylated H3K36Me3 and H3K4Me3, but only a non-significant decrease in H3K79Me3 (Fig. 5A). Hcy supplementation had no effect on H3K79Me2 and a weak, non-significant effect on H3K36Me2 (Fig. 5A). In accordance, the H3K79Me2 levels in the Δ*sah1* mutant were unaltered; however, the H3K36Me2 levels were significantly decreased (Fig. 5A). Noteworthily, Hcy-supplemented wild type also exhibited significantly increased levels of H3K79Me1 compared to those in non-supplemented wild-type yeast (Fig. 5A). Accordingly, a non-significant trend to elevated H3K79Me1 levels was also observed in the Δ*sah1* mutant (Fig. 5A).

H3 methylation levels in the *Drosophila* Hcy supplementation model were not affected compared to those in controls in any of the tested histone methylation sites (Fig. 5B). In contrast, H3 methylation in migratory L3 larvae of the SAHH genetic model was decreased at almost all the tested sites, with H3K4Me3, H3K36Me2 and H3K79Me1 being significantly decreased, H3K36Me3 and H3K79Me2 being non-significantly decreased, and H3K79Me1 being unaffected (Fig. 5B). We conclude that although SAH inhibits H3 lysine methylation in both yeast and *Drosophila*, the extent of inhibition varies across different methylation sites. This further supports the hypothesis that SAH accumulation selectively inhibits distinct protein methylation processes and necessitates functional characterization of evolutionary conserved SAH-dependent methylation targets responsive to Hcy.

## DISCUSSION

The mechanisms by which elevated Hcy leads to CVD are not yet fully understood. SAH, a strong product inhibitor of SAM-dependent methyltransferases and a more sensitive indicator of CVD than Hcy (Kerins et al., 2001), accumulates in response to elevated Hcy and is proposed to be causally involved in cardiovascular remodeling (Cacciapuoti, 2013). Although the dietary and genetic *Drosophila melanogaster* fly and *Saccharomyces cerevisiae* yeast models developed here show elevated SAH and share most of the defects in growth, development and methylation, the severity of the phenotypes often varies between the dietary and genetic models. In particular, growth and developmental defects are more severe in the genetic models. Specifically, compared to the strong growth inhibition and cell death observed upon SAHH deletion in yeast, Hcy-supplemented wild-type yeast exhibited less pronounced growth inhibition and no reduction in survival. Similarly, *Drosophila* SAHH knockdown larvae showed massively reduced survival at both pupariation and adult hatching, accompanied by drastically delayed pupariation and hatching, whereas Hcy-supplemented *Drosophila* larvae showed a more modest developmental delay, successful pupariation and normal adult hatching.

Two explanations could account for the more detrimental phenotypes of the genetic compared to the Hcy-supplementation models: (1) the higher SAH accumulation in the genetic models, or (2) the inability to synthesize Hcy in the SAHH mutants. The latter is unlikely in yeast, which can produce Hcy even in the absence of SAHH, via the yeast-specific sulfur assimilation pathway (Visram et al., 2018). Consequently, the 2-fold higher SAH levels in the *Δsah1* mutant compared to Hcy-supplemented wild type are likely to be the reason for the stronger defects. Of note, disruption of this alternative Hcy synthesis pathway in the *Δsah1* mutant leads to inviability of the respective double mutant, which can be rescued by Hcy supplementation (Visram et al., 2018). In contrast, *Drosophila* cannot produce Hcy via an alternative pathway. Thus, because both glutathione synthesis and the folate cycle require Hcy (Tehlivets et al., 2013; Elmore et al., 2007), their impairment may contribute to the more severe phenotype in the genetic versus dietary fly models.

Both the yeast and fly dietary models accumulate SAH without concomitant SAM accumulation, resulting in decreased SAM/SAH ratios. In contrast, the genetic models accumulate both SAH and SAM, with little or no effect on SAM/SAH ratio. SAM is synthesized in the methylation pathway after remethylation of Hcy to methionine. However, Hcy production is impaired in *Drosophila* with SAHH-encoding gene knockdown. Therefore, other mechanisms must be responsible for the SAM buildup in this model. In addition to upregulation of SAM synthesis from methionine by methionine-adenosyltransferase (MAT), both the yeast and *Drosophila* models are expected to accumulate SAM, owing to reduced consumption by SAM-dependent methyltransferases because of SAH-mediated inhibition and/or SAM sparing, suggesting an additional adverse effect on methylation (Ye et al., 2017, 2019; Ye and Tu, 2018). Supporting SAM trapping in response to a block in SAH hydrolysis, human patients deficient in SAHH accumulate both SAM and methionine (Baric et al., 2004). Noteworthily, SAM at high concentrations was shown to have a direct inhibitory effect on methylation via the production of adenine, an endogenous SAHH inhibitor (Fukumoto et al., 2022), and methylthioadenosine, another metabolite produced during catabolism of SAM by the methionine salvage pathway, which was shown to inhibit directly protein arginine methyltransferase (PRMT)5-mediated symmetric dimethylation of H4R3 (Kryukov et al., 2016), as well as to be highly toxic to dividing cells (Fukumoto et al., 2022). This suggests further mechanisms that might explain the more severe phenotypes of the fly and yeast genetic models compared to those of the dietary models. However, given that the fly dietary and genetic models showed similar developmental delays, along with an accumulation of SAH, the growth defects in response to Hcy are likely due to deficient methylation rather than other effects.

It has to be noted that, in contrast to yeast, it is unclear which fly tissues/organs are exposed to the supplemented Hcy or its metabolites in the dietary model. Similarly, based on transcriptome data (Chintapalli et al., 2007) endogenous SAHH varies between fly organs. While the global RNA knockdown efficiency is 70%, interference with *in vivo* SAHH function may vary between tissues/organs dependent on endogenous expression levels and organ-specific knockdown efficiencies. Thus, comparing the dietary and genetic models in flies is more complex than in yeast and needs further exploration, e.g. of the tissue-specific RNAi expression.

SAH is known to inhibit SAM-dependent methyltransferases, although the sensitivity of individual enzymes to this inhibition differs (Esse et al., 2013; Gaynor and Carman, 1990; Yao et al., 2011). We therefore examined the effects of SAH on different methylation reactions, including PL methylation. In yeast, PE is converted to PC via three sequential methylation steps, with the first one catalyzed by Cho2, and the second and third one by Opi3 (Ye et al., 2017). Accumulation of PE, accompanied by reduced MMPE, DMPE and PC, in Hcy-supplemented wild-type yeast suggests inhibition of Cho2 and possibly Opi3 by SAH. Interestingly, in the yeast Δ*sah1* mutant, changes in PL methylation intermediates were much less dramatic. A potential explanation could be the high SAM levels in Δ*sah1*, which might buffer the effects of increased SAH by shifting the competitive favor in the direction of SAM. Alternatively, higher elevation of SAH in the yeast Δ*sah1* mutant can potentially induce different compensatory mechanisms to preserve membrane structure and function, such as synthesis of PC from choline and/or PL remodeling (Boumann et al., 2003). In line, PL methylation and synthesis of PC from choline were shown to produce different PL species (Boumann et al., 2004).

Although PL methyltransferase activity has been detected in *Drosophila* (de Sousa et al., 1988), the exact mechanisms remain unknown*.* The accumulation of PE, concomitant with reduced MMPE levels in the fly genetic model, suggests that the first methylation step is inhibited. In contrast, in the fly dietary model, MMPE accumulates whereas DMPE is reduced, suggesting that the second methylation step is primarily inhibited. These results suggest differential SAH sensitivity of the enzymes catalyzing the first and second step of PE to PC methylation in flies. Detection of MMPE and DMPE intermediates, as well as changes in their levels and accumulation of PE in our experiments, support the existence of PL methylation in *Drosophila*. Despite the reduction of these intermediates, PC levels were not significantly reduced in flies, neither in the dietary nor the genetic model, likely due to methylation-independent synthesis of PC from choline.

In addition to PL methylation, we investigated protein methylation, focusing on global protein arginine methylation, as well as on lysine methylation of histones. Methylation of arginine is catalyzed by PRMTs, which are highly evolutionarily conserved between yeast and *Drosophila* up to mammals (Wang and Li, 2012). Specifically, SAH was shown to inhibit PRMT1, which shows high sequence similarities to the yeast Rmt1 (70%; also known as Hmt1) and *Drosophila* DART1 (84%; also known as Art1) (Wang and Li, 2012; Qian et al., 2018). PRMT1, Rmt1 and DART1 are the predominant PRMTs in these species and methylate arginine in two steps, first generating MMA, which is then further methylated, resulting in ADMA. Because both Hcy-supplemented wild-type yeast and the yeast Δ*sah1* mutant showed significantly decreased ADMA/Arg ratios despite different SAM/SAH ratios, inhibition of protein arginine dimethylation is likely to be better reflected by the levels of SAH accumulation than by SAM/SAH ratio. A similar observation was previously made for DNA methylation in mammals, in which SAH proved to be a better proxy for inhibition than the SAM/SAH ratio (Caudill et al., 2001; Mandaviya et al., 2014). In contrast to yeast, the ADMA/Arg ratio was unaltered in the fly dietary model, despite elevated SAH levels. However, although no global effects were observed, this does not exclude that arginine methylation of some individual proteins is affected. Indeed, the observation of both increased and decreased signals detected with anti-MMA antibody in yeast and flies in dietary as well as genetic models points to a potential differential effect of SAH-mediated methylation inhibition on different proteins. Arginine methylation, specifically on histones 3 and 4, plays an important role in regulating the development of *Drosophila* (Cakouros et al., 2008; Kimura et al., 2008). Interestingly, mutations in the DART1-encoding gene were reported to lead to low viability at pupal stage and strongly delayed development (Kimura et al., 2008). Moreover, DART1 was shown to dimethylate arginine 3 on histone 4 (H4R3Me2) and to interact with the nuclear ecdysone receptor, which is indispensable for normal development in *Drosophila* (Kimura et al., 2008), suggesting that DART1 inhibition may be involved in the developmental delay observed in response to Hcy supplementation in flies and/or developmental arrest in the fly genetic model, despite unaffected total ADMA/Arg ratio in the fly dietary model.

Although our data suggest that, at least in yeast, total arginine methylation is affected to similar extents in the dietary and genetic models, histone lysine methylation tends to be more affected in the genetic than in the dietary models in both yeast and *Drosophila*. However, analysis of protein lysine methylation of H3 showed different extents of inhibition of different types of lysine methylations in yeast and flies. In particular, in yeast, H3K36 trimethylation was inhibited stronger in response to Hcy than to dimethylation at the same site. Similarly, H3K79 monomethylation, dimethylation and trimethylation were differently altered in response to Hcy and SAH in yeast and in *Drosophila*, which further suggests that the inhibitory capacity of SAH might differ for various protein methylation reactions. Noteworthily, H3K4Me3, inhibited in yeast as well as the fly genetic model, was reported to be decreased in association with elevated SAH (Fukumoto et al., 2022).

Histone methylation, another major consumer of methyl groups, is catalyzed either by SET domain lysine methyltransferases, e.g. H3K4 and H3K36 methylations, or by lysine methyltransferase without a SET domain (Dot1 in yeast or Grappa in *Drosophila*) such as H3K79 methylation (Ye et al., 2017; Del Rizzo and Trievel, 2011; Shanower et al., 2005). The highly diverged catalytic properties reported for Dot1 methyltransferases may explain the different inhibitory potential of Hcy and SAH toward H3K79 in our models compared to those of histone lysine methylation sites methylated by SET domain-containing methyltransferases (Stulemeijer et al., 2015). However, the stronger effect of genetic models on protein methylation suggests unexpected complexity of SAH-mediated inhibition of methyltransferases that needs further exploration. Noteworthily, decreased H3K36 trimethylation may be due to active demethylation, which is regulated by demethylation of PP2A, rather than direct inhibition of histone methylation (Ye et al., 2019).

Furthermore, *Drosophila* SAHH knockdown larvae exhibited markedly reduced size, similarly to significantly reduced weight of larvae grown on Hcy-containing food and consistent with the severe effect of global SAHH knockdown on larval viability; the downregulation of SAHH inhibitors was also shown to increase lifespan in *Drosophila* (Parkhitko et al., 2016). In addition to decreased size, delayed development and poor viability, *Drosophila* SAHH knockdown larvae exhibited black spots characteristic of melanotic tumors. Although formation of melanized bodies was reported in association with various defects, it was in particular shown to be linked to histone 4 lysine 20-specific methyltransferase SET8 (Fang et al., 2002; Minakhina and Steward, 2006) and *Drosophila* DNA methyltransferase 2 (dDnmt2; also known as Mt2), an ortholog of human DNMT2 (also known as TRDMT1) catalyzing methylation of DNA and tRNAs (Lin et al., 2005; Schaefer et al., 2010; Durdevic et al., 2013). Furthermore, it has been shown that Hox genes, which are regulated via H3 lysine methylation, are also associated with the formation of melanized bodies (Ponrathnam et al., 2021; Jambhekar et al., 2019; Paco et al., 2020; Chen and Armstrong, 2015). Interestingly, dysregulation of Hox genes was reported to result not only in the formation of melanized (pseudo-) tumors but also to lead to pupal lethality in *Drosophila* (Ponrathnam et al., 2021), which is in line with our observation of strongly decreased survival rates, especially at pupal stage, in *Drosophila* SAHH knockdown flies.

In summary, comparison of *Drosophila* and yeast models of Hcy and SAH accumulation to reveal evolutionarily conserved methylation targets responsive to inhibition by SAH in association with elevated Hcy showed similar as well as different methylation patterns and suggests that SAH is a better indicator of methylation deficiency than SAM/SAH ratio. Furthermore, the observation of both PL and protein methylation inhibition in our models supports the involvement of multiple mechanisms in pathological consequences associated with elevated Hcy and/or SAH. Employing yeast and *Drosophila* genetic screens will be a promising future strategy to pinpoint evolutionarily conserved Hcy/SAH-dependent mechanisms with high potential relevance for HHcy-associated human pathologies.

## MATERIALS AND METHODS

### Chemicals and consumables

All used chemicals and consumables, including their sources, are provided in Dataset 1.

### Yeast strains, media and growth conditions

*Saccharomyces cerevisiae* strains used in this study are congenic with BY4741, a derivative of S288C, and are listed in Table S1. Cells were grown at 30°C in synthetic dextrose complete medium (SDC) containing 1.4 g/l Difco yeast nitrogen base, 5 g/l ammonium sulfate, 20 g/l glucose and 0.79 g/l complete supplement mixture (CSM) at 180 rpm in the absence or presence of Hcy, as indicated in individual experiments. Media were solidified by the addition of 20 g/l agar.

For growth curves, yeast wild-type cells were grown overnight in SDC medium and inoculated to optical density at 600 nm wavelength (OD_600_)=0.05 in fresh SDC medium containing 0, 1, 2 or 5 mM Hcy. The Δ*sah1* mutant cells were grown overnight in SDC medium and inoculated to OD_600_=0.05 in fresh SDC medium without Hcy. 300 µl of each cell suspension was applied into a honeycomb well plate in three technical replicates. OD_600_ was measured every 30 min for 40 h in a Bioscreen C (Dynex) with constant shaking between measurements. Before each measurement, shaking was stopped for 5 s. For cell death analysis, measurement was paused after 30 h, and, per sample, 20 µl was transferred to a round-bottom 96-well plate. 180 µl PI (100 ng/ml in PBS) or 180 µl PBS was added and incubated for 5 min at room temperature (RT) in the dark. The plate was centrifuged for 5 min at 1600 ***g*** and RT, the supernatant was removed, and cells were resuspended in 200 µl PBS. For quantification of PI-positive (dead) cells, samples were measured using flow cytometry (BD LSRFortessa; 561 nm excitation, 610/20 nm emission). Per sample, 30,000 events were evaluated and analyzed with BD FACSDiva software, with gating on singlet cells using forward scatter area versus height signals and setting PI-positive gates using unstained controls. Additionally, 30 h cell size was measured on a cell counter CASY (Innovatis). To this end, 100 µl per sample was taken, diluted 1:5000 in CASY buffer (0.9% NaCl+1 mM EDTA in ddH_2_O) and transferred into a Transsonic T460 ultrasound bath (Elma) for 30 s before cell size analysis. After 40 h of growth curve analysis, samples were taken for microscopy. For microscopic observation, live cells were immobilized using agar sheets (Wolinski and Kohlwein, 2015). Transmission images were acquired using a Leica SP5 confocal microscope (Leica Microsystems, Inc.), a 488 nm argon laser line and a HC PL APO 63×/1.4 NA oil immersion objective. The experiment was independently repeated once (Dataset 4).

### *Drosophila melanogaster* lines, fly food and breeding

Fly lines used in this study are listed in Table S2. Fly lines generated in this study are available on request to R.P.K.’s laboratory. FlyBase (Jenkins et al., 2022) was consulted for experimental planning. Flies were propagated in 68 ml *Drosophila* containers equipped with mite-tight stoppers on standard fly food containing 15.7 g/l baker's yeast, 8.7 g/l soy flour, 5.4 g/l agar-agar, 69.6 g/l maize flour, 19.1 g/l beet syrup, 69.6 g/l malt, 5.4 ml/l propionic acid and 1.3 g/l methyl-4-hydrobenzoate (dissolved in 4.4 ml EtOH). If not mentioned otherwise, stock keeping and fly experiments were done at 25°C and 70% humidity with 12 h light/12 h dark cycle.

For Hcy-supplemented food, 5% (v/v; in water) 400 mM DL-Hcy was added to fly food prior to solidifying. For control food, 5% water was added instead of Hcy. Eggs from Canton-S or *w[1118]* flies were collected on apple juice agar plates [2.125% (w/v) agar, 20% (v/v) apple juice, 2% (w/v) sugar, 0.12% (w/v; dissolved in EtOH) nipagin] supplemented with baker's yeast paste. Agar pieces with 100 eggs each were cut out and transferred onto either standard or Hcy-supplemented fly food. Vials were kept at 25°C at 70% humidity. Four individual vials were prepared for each food. From each vial, ten migratory L3 larvae were collected, rinsed with water, dried with blotting paper and weighed individually on a Sartorius MC 5 scale. Larvae were frozen at −80°C in batches of five larvae for SAH and SAM extraction, protein extraction and PL analysis. Remaining larvae were kept at 25°C and 70% humidity, and the number of pupae and empty pupae cases were counted continuously until no further pupae appeared or hatched. The experiment was independently repeated once (Dataset 4).

For RNAi crosses, per vial, ten adult virgin female flies carrying ubiquitous driver transgene were crossed to five male flies carrying either an mCherry RNAi (RNAi control) or an SAH hydrolase RNAi (SAHH RNAi) transgene in standard food vials and incubated for 24 h at 25°C and 70% humidity. After incubation, adult flies were flipped to new vials, and eggs were counted. New vials were incubated again for 24 h before adult flies were removed and eggs were counted. Four individual vials were prepared for each crossing and were kept at 25°C and 70% humidity. The numbers of pupae and empty pupae cases were counted continuously in two vials per crossing. The experiment was independently repeated once. Larvae from the other two vials were extracted, rinsed and photographed daily for up to 10 days. Photographs were taken on a Leica M60 binocular with a Google Pixel 6a smartphone. For RNAi control, migratory L3 larvae were collected for all further analyses. For SAHH RNAi, migratory L3 larvae were collected for analysis of total protein arginine methylation, histone lysine methylation and analysis of SAHH expression. As the majority of larvae globally expressing SAHH RNAi never started to migrate, we collected 14-day-old non-migratory larvae for analysis of SAH, SAM and PLs. Upon collection, all larvae were rinsed with water, dried with blotting paper, weighed in batches of five larvae and frozen at −80°C for reverse transcription quantitative PCR (RT-qPCR), SAH and SAM extraction, protein extraction and PL analysis.

The identity of organ-specific driver lines (see Table S2) was confirmed by crossing three virgin females of the GFP-reporter line (see Table S2) to three males of the driver lines. F1 migratory L3 larvae were rinsed with and mounted in cold water before imaging using a Leica M165 FC fluorescence stereomicroscope equipped with a GFP emission filter and a Leica DFC3000 G camera. To score survival and developmental delay in response to organ-specific SAHH knockdown (see Table S2) compared to those in mCherry RNAi controls (see Table S2), five virgin females of each of the effector lines were crossed to three to four organ-specific or ubiquitous driver males. Parental flies were flipped daily three to four times, and egg numbers as well as eclosed adult flies (based on empty pupal cases) were counted to assess hatching rates. To score developmental delay, the time to adult eclosion was assessed daily after day 11 post crossing for balancer-carrying (control) versus non-balancer-carrying (fat body-specific driver) progeny of the heterozygous fat body-targeting driver line crossed to flies carrying the SAHH RNAi transgene (see Table S2). The experiment was repeated with independent parental flies, and progeny of three to four consecutive daily collections were scored (Dataset 4).

### *Drosophila* food intake

1% (w/v) Brilliant Blue was added to fly food containing either 5% (v/v; in water) 400 mM DL-Hcy or 0.2% (w/v) caffeine and 5% (v/v) water. Fly food with 1% (w/v) Brilliant Blue and 5% (v/v) water was used as a control. Colored fly food was poured into small Petri dishes to cover the bottom. 25 male and 25 female adult flies were crossed in standard food vials and flipped to new vials every 24 h. All vials were kept at 25°C and 70% humidity. Larvae were extracted from food vials 1 day prior to reaching the migratory stage, rinsed and transferred to colored food plates. Plates were incubated for 1 h in darkness at RT before transferring the plates to ice. Larvae were extracted from food plates, rinsed, collected in cohorts of ten larvae into 2 ml save-seal tubes and weighed on a Sartorius MC 5 scale. After adding 350 µl MeOH and one 5 mm steel ball, samples were homogenized in a Retsch MM40 homogenizer at 30 Hz and 4°C for 2 min. Homogenates were centrifuged for 5 min at 20,000 ***g*** and 4°C in a 5430 R centrifuge (Eppendorf), and the supernatant was transferred to a new vial and centrifuged again for 5 min at 20,000 ***g*** and 4°C. Duplicates of 100 µl supernatant were aliquoted into a 96-well plate, and absorbance was measured at 626 nm in a Spectrostar^Nano^ spectrophotometer (BMG Labtech). For absolute quantification, a five-point calibration curve using colored fly food (0.5–7 mg) after extraction with MeOH was prepared as described above. The experiment was independently repeated once (Dataset 4).

### RT-qPCR

SAHH primers were selected from the FlyPrimerBank of the Fly RNAi Database. *Gapdh1/2* primers were selected according to Beaucher et al. (2007) and are listed in Table S3. RNA from migratory L3 larvae ubiquitously expressing either mCherry RNAi or SAHH RNAi was extracted using RNeasy Minikit according to the manufacturer's instructions for animal tissues. RNA concentration was determined by Nanodrop ND-1000 (Peqlab). 1 µg RNA was treated with DNAse I and reverse transcribed using the Invitrogen superscript III One-Step RT-PCR System according to the manufacturer's instructions. For quantitative PCR, 8 ng cDNA, 0.1 nmol forward and reverse primers (Table S3), 10 µl Taq Universal SYRB Green Supermix and water to reach a final reaction volume of 20 µl were used and analyzed on a StepOne Plus RT-PCR system (Applied Biosystems). *Gapdh1/2* were used as housekeeping genes (see Table S3) for normalization. Results were calculated in Excel (Office Professional Plus 2021; Microsoft) according to Schmittgen and Livak (2008) and depicted as 2^-ΔCt^ values. All samples were measured in two biological replicates, each biological replicate in two technical replicates.

### SAH/SAM extraction and analysis

Extraction of SAH and SAM was based on Gellekink et al. (2005) with minor changes. Briefly, for yeast, per sample, 20 OD_600_ units were harvested and resuspended in 250 µl water+0.1% formic acid. After addition of 250 µl glass beads, cells were lyzed in Homogenizer MM40 (Retsch) at 30 Hz and 4°C three times for 40 s. Homogenates were diluted 1:2. For *Drosophila*, migratory L3 larvae were used for the dietary model as well as RNAi control, and, as the majority of larvae globally expressing SAHH RNAi never started to migrate, 14-day-old non-migratory larvae were used for SAHH RNAi. Per sample, five larvae were put into 500 µl water+0.1% formic acid and homogenized with a steel ball in Homogenizer MM40 (Retsch) at 30 Hz and 4°C for 2 min. For SAH and SAM extraction, 1 ml Bond Elut PBA columns (100 mg bed mass) were used. Solid-phase extraction was performed after washing the columns with 4 ml 0.1 M formic acid and equilibrating with 4 ml 20 mM ammonium acetate buffer (pH 7.4). Two separate aliquots of 210 µl were prepared for SAH and SAM extraction, respectively. Per aliquot, 60 µl internal standard was added (either 2 µM SAH-d4 or 5 µM SAM-d3 in water) and 90 µl of the mix was applied onto the column. The columns were subsequently washed with 3 ml 20 mM ammonium acetate butter (pH 7.4). Samples were eluted in 1 ml 0.1 M formic acid and measured directly via high-performance liquid chromatography with triple-quadrupole tandem mass spectrometry. Per sample two technical replicates were analyzed.

SAH and SAM levels were analyzed by a 1290 Infinity UHPLC coupled to a 6470 Triple-Quadrupole mass spectrometer (Agilent) using a BEH C18 column (3.0 mm×150 mm; 1.7 μm) with 50°C column temperature, 5 μl injection volume and a constant flow rate of 200 μl/min. H_2_O+0.1% formic acid (solvent A) and MeOH+0.1% formic acid (solvent B) were used as solvents. 95% solvent A was held for 2 min, followed by a change to 100% solvent B over the next 2 min, which was held for an additional 3.5 min. Re-equilibration was carried out by changing to 95% solvent A within 5 s, followed by 3 min at 95% solvent A. Total run time was 11 min. For absolute quantification, independent SAH and SAM dilution series were prepared for eight-point calibration curves in the range from 1.56 to 200.0 nM. All analytes were measured in MRM mode with dwell time of 50 ms and cell acceleration voltage of 4 V for all transitions. Fragmentor voltage was optimized for each transition individually and set between 97 and 115 V. The transitions 385.1−135.9 m/z for SAH with a collision energy of 15 eV and 389.1–137.9 m/z for SAH-d4 with a collision energy of 21 eV were used as quantifiers. The transitions 385.1–133.9 m/z (collision energy 15 eV) and 87.9 (collision energy 40 eV) were the qualifiers for SAH, and 389.1–91.9 m/z with a collision energy of 40 eV were the qualifiers for SAH-d4. The transitions 399.2–250.0 m/z for SAM with a collision energy of 15 eV and 402.2–249.9 m/z for SAM-d3 with a collision energy of 13 eV were used as quantifiers. The transitions 399.2–135.9 m/z (collision energy 27 eV) and 96.9 (collision energy 35 eV) were the qualifiers for SAM, and 402.2–135.8 m/z and 96.9, both with a collision energy of 33 eV, were the qualifiers for SAM-d3.

### Total protein ADMA/Arg ratio

For total protein ADMA and arginine analysis, yeast (15 OD_600_ units) and *Drosophila* (ten migratory L3 Canton-S larvae) samples were suspended in 400 μl ice-cold MeOH and 200 μl Milli-Q H_2_O, and transferred to Precellys tubes with 1.4 mm diameter zirconium oxide beads. This suspension was homogenized two times for 20 s by a Precellys 24 tissue homogenizer at 25°C. Afterwards, the homogenized samples were centrifuged at 10,600 ***g*** for 30 min at 4°C and stored at −20°C for at least 2 h. Precipitates were further processed for arginine methylation analyses as described in Zhang et al. (2021) and Habisch et al. (2021). Briefly, the precipitates were hydrolyzed with 6 M HCl to obtain amino acids and lyophilized. For nuclear magnetic resonance (NMR) analysis, dried samples were re-dissolved in 500 μl NMR buffer [0.08 M Na_2_HPO_4_, 5 mM 3-(trimethylsilyl) propionic acid-2,2,3,3-d4 sodium salt (TSP), 0.04 (w/v)% NaN_3_ in D_2_O, pH adjusted to 7.4 with 8 M HCl and 5 M NaOH]. NMR experiments were carried out as described by Zhang et al. (2021) and Habisch et al. (2021). 2D JRES (^1^H homo-nuclear J-resolved spectroscopy) spectra were acquired at 310K on a Bruker 600 MHz Avance Neo spectrometer equipped with a TXI 600S3 probe head using the jresgpprqf pulse sequence [16 scans, size of free induction decay 16,384 (direct dimension F2)/256 (indirect dimension F1), 10,000.00/78.042 Hz spectral width in F2 (chemical shift axis)/F1 (spin–spin coupling axis), recycle delay 2 s] with presaturation during the relaxation delay to obtain virtually decoupled spectra. Data were processed in Bruker Topspin version 4.3 using the SINE and QSINE window functions (Shifted Sine Bell value=0) in F2/F1. Fourier transform was performed with 16,384/256 F2/F1 points of the fid. 2D J-resolved experiments were processed using back prediction implemented in the Bruker AU program proc_jres.be (Gellekink et al., 2005). The JRES spectra were then projected along F2 and exported as 1D NMR spectra. Quantification of arginine and ADMA was carried out by integration of characteristic peaks as described elsewhere (Habisch et al., 2021; Zhang et al., 2021).

### Protein extraction

For yeast, total proteins were extracted from 1 OD_600_ unit per sample. Per condition, four biological replicates were analyzed. For extraction, pellets were resuspended in 200 µl 1.85 M NaOH with 7.5% (v/v) β-mercaptoethanol and incubated for 10 min on ice. 200 µl 50% trichloroacetic acid was added, and samples incubated for a further 10 min on ice. Samples were then centrifuged for 20 min at 20,000 ***g*** and 4°C, supernatant removed and samples centrifuged again for 2 min. Pellets were washed twice, centrifuged for 5 min at 20,000 ***g*** and 4°C, and supernatant was removed. Pellets were then resuspended in 100 µl sample buffer [0.156 M Tris-HCl, 5% SDS, 20% glycerol (87%), 0.01% Bromophenol Blue, 300 µM DTT], and samples were stored at −20°C.

For *Drosophila*, total proteins were extracted from five migratory L3 larvae (Canton-S for the dietary model and larvae globally expressing mCherry RNAi or SAHH RNAi for the genetic model) per sample. Per condition, four biological replicates were analyzed. Per sample, 100 µl extraction buffer [9.75 ml PBS (pH 7.4)+50 µl Triton X-100+200 µl 0.1 M (in acetone) PMSF] and one 5 mm steel ball were added, and the sample was homogenized in a Retsch MM40 homogenizer at 30 Hz and 4°C for 2 min. Samples were incubated for an additional 10 min at 4°C on an overhead rotator SB3 (Stuart). Afterwards, the homogenate was transferred to a new vial and centrifuged for 10 min at 6500 ***g*** and 4°C in a 5430 R centrifuge (Eppendorf). The supernatant was removed, and the pellet was resuspended in 100 µl 0.4 M HCl followed by 5 min incubation on ice. Samples were then centrifuged for 10 min at 6500 ***g*** and 4°C. The supernatant was transferred to a new vial, and pH was set to 7 with 1 M NaOH. The sample was mixed with equal volume of sample buffer (0.3125 M Tris-HCl, 10% SDS, 40% glycerol, 0.02% Bromophenol Blue, 600 µM DTT) and stored at −20°C.

### Western blotting

Polyacrylamide gel electrophoresis (PAGE) was performed using Invitrogen NuPAGE (12% BT 1.0) gels. Per well, 5 µl sample was applied and consecutively adjusted to equalize signals of normalization antibody. After PAGE, proteins were blotted onto polyvinylidene fluoride (PVDF) Immobilon-P Transfer Membrane (0.45 µm). Western blot analysis was performed using the following antibodies, with given dilutions for yeast and *Drosophila* samples, respectively: anti-Gapdh antibody (1:14,000 or 1:5000), anti-MMA antibody (1:1000 or 1:200), anti-ADMA antibody (1:1000 or 1:200), anti-H3 antibody (1:5000 or 1:1000), anti-H3K4Me3 antibody (1:1000 or 1:200), anti-H3K36Me2 antibody (1:1000 or 1:200), anti-H3K36Me3 antibody (1:1000 or 1:200), anti-H3K79Me1 antibody (1:1000 or 1:200), anti-H3K79Me2 antibody (1:1000 or 1:200), anti-H3K79Me3 (1:500 or 1:100) and secondary anti-rabbit horseradish peroxidase-conjugated antibody (1:15,000). Sources for all antibodies are provided in Dataset 1.

For immunostaining, membranes were blocked in 2% (w/v) skim milk in TST buffer [0.05 M Tris-HCl, 0.15 M NaCl, 0.1% (v/v) Tween-20, pH 7.4] for 1 h at RT. Primary antibody was diluted in 1% (w/v) skim milk in TST buffer, and membranes were incubated in the primary antibodies for 1 h at RT. Membranes were then washed three times for 5 min in TST buffer at RT. Secondary antibody was diluted in 1% (w/v) skim milk in TST buffer, and membranes were incubated in secondary antibody for 1 h at RT. Membranes were then washed three times for 5 min in TST buffer at RT. Protein signals were detected using a Clarity Western ECL Substrate Kit and captured with a ChemiDoc Touch Imaging System (Bio-Rad). Quantification was done in ImageLab Software Version 6.0.1 (Bio-Rad). Between different immunostainings, primary/secondary antibodies membranes were regenerated by incubation in stripping buffer [2% (v/v) SDS, 0.064 M Tris-HCl, 0.7% (v/v) β-mercaptoethanol] for 20 min at 60°C before the next immunostaining. The experiment was independently repeated once (Dataset 4). Full blots are shown in Dataset 5.

### PL methylation

Lipids were extracted from five *Drosophila* L3 larvae (migratory L3 Canton-S larvae for dietary model and migratory L3 RNAi control larvae, as the majority of larvae globally expressing SAHH RNAi never started to migrate, 14-day-old non-migratory larvae for SAHH RNAi for the genetic model) or 20 OD_600_ units in yeast per sample according to the protocol described in Matyash et al. (2008). Briefly, 700 µl MTBE:MeOH (10:3, v/v) and 50 µl internal standard mix [yeast: PC 38:0, PE 34:0; *Drosophila*: PC 38:0; 0.2 mg/ml in MTBE:MeOH (10:3; v/v)] were added to larvae or yeast cell pellets with either a 5 mm steel ball (*Drosophila*) larvae or 250 µl glass beads (yeast) in 2 ml safe-seal tubes. Samples were homogenized in a Homogenizer MM40 (Retsch) at 30 Hz and 4°C for 5 min. 200 µl water (MS grade) was added and mixed in the Homogenizer MM40 at 30 Hz and 4°C for a further 5 min. The upper phase was collected, and a second extraction was performed by adding 700 µl MTBE:MeOH (10:3, v/v), mixing in the Homogenizer MM40 (Retsch) at 30 Hz and 4°C for another 5 min, and the upper phase was pooled with the first extraction. Solvent was removed under a nitrogen stream at 38°C. For measurement, samples were resuspended in 1 ml isopropanol with 10 mM ammonium acetate, 0.1% formic acid and 8 µM phosphoric acid.

PLs were analyzed by a 1290 Infinity UHPLC coupled to a 6470 Triple-Quadrupole mass spectrometer (Agilent) using a BEH C18 column (3.0 mm×150 mm; 1.7 μm) with 50°C column temperature, 5 μl injection volume and a constant flow rate of 200 μl/min. H_2_O+10 mM ammonium acetate+0.1% formic acid+8 µM phosphoric acid (A) and isopropanol+10 mM ammonium acetate+0.1% formic acid+8 µM phosphoric acid (B) were used as solvents. 50% solvent A was held for 0.5 min, followed by a change to 80% solvent B over the next 8.5 min and a change to 100% solvent B over next 13 min. 100% solvent B was held for 2.5 min. Re-equilibration was carried out by changing to 50% solvent A within 0.5 min, which was held for 5 min. Total run time was 30 min. All analytes were measured in dynamic multiple reaction monitoring mode with optimized individual retention times, retention window of 4 min and cell acceleration voltage of 5 V for all transitions. Fragmentor voltage and collision energy was individually optimized for each lipid class individually. A full list with all analyzed transitions, including individual retention times, fragmentor voltage and collision energy, is shown in Dataset 2.

### Figure preparation and statistics

All diagrams (bar charts, *xy*-charts and box plots) were prepared in Prism 8 (GraphPad). All collages (yeast and *Drosophila* photographs, western blots and figure blocks) were compiled in PowerPoint (Office Professional Plus 2021; Microsoft). All statistics were calculated in SPSS 27.0 (SPSS Inc.). Datasets were checked for normal distribution by Shapiro–Wilk test. If not normally distributed (non-parametric), significances were calculated by two-tailed Mann–Whitney *U*-test for single comparisons and two-tailed Kruskal–Wallis test with Bonferroni correction for multiple testing. If normally distributed (parametric), homogeneity of variance was checked by Levene test. In case of homogenous variances, significances were calculated via unpaired two-tailed Student’s *t*-tests for single comparisons, and two-tailed one-way ANOVA with Bonferroni correction for multiple testing or Games-Howell correction for multiple testing for results with non-homogenous variances. All reported *P*-values are two sided, with an α-level of 0.05. If applicable, parametric significances are marked by asterisks (*) and non-parametric significances are marked by hashtags (^#^). All results of statistical analyses are shown in Dataset 3.

### Use of AI tools

No AI tools were used for data analysis or writing of the manuscript.

## Supplementary Material

10.1242/dmm.052802_sup1 Supplementary information

## References

[DMM052802C1] Agha, G., Mendelson, M. M., Ward-Caviness, C. K., Joehanes, R., Huan, T., Gondalia, R., Salfati, E., Brody, J. A., Fiorito, G., Bressler, J. et al. (2019). Blood leukocyte DNA methylation predicts risk of future myocardial infarction and coronary heart disease. *Circulation* 140, 645-657. 10.1161/CIRCULATIONAHA.118.039357 31424985 PMC6812683

[DMM052802C2] Alani, H., Tamimi, A. and Tamimi, N. (2014). Cardiovascular co-morbidity in chronic kidney disease: current knowledge and future research needs. *World J. Nephrol.* 3, 156-168. 10.5527/wjn.v3.i4.156 25374809 PMC4220348

[DMM052802C3] Alter, P., Rupp, H., Rominger, M. B., Figiel, J. H., Renz, H., Klose, K. J. and Maisch, B. (2010). Association of hyperhomocysteinemia with left ventricular dilatation and mass in human heart. *Clin. Chem. Lab. Med.* 48, 555-560. 10.1515/CCLM.2010.102 20148720

[DMM052802C4] Baric, I., Fumic, K., Glenn, B., Cuk, M., Schulze, A., Finkelstein, J. D., James, S. J., Mejaski-Bosnjak, V., Pazanin, L., Pogribny, I. P. et al. (2004). S-adenosylhomocysteine hydrolase deficiency in a human: a genetic disorder of methionine metabolism. *Proc. Natl. Acad. Sci. USA* 101, 4234-4239. 10.1073/pnas.0400658101 15024124 PMC384724

[DMM052802C5] Beaucher, M., Hersperger, E., Page-McCaw, A. and Shearn, A. (2007). Metastatic ability of Drosophila tumors depends on MMP activity. *Dev. Biol.* 303, 625-634. 10.1016/j.ydbio.2006.12.001 17239363

[DMM052802C6] Bonaa, K. H., Njolstad, I., Ueland, P. M., Schirmer, H., Tverdal, A., Steigen, T., Wang, H., Nordrehaug, J. E., Arnesen, E., Rasmussen, K. et al. (2006). Homocysteine lowering and cardiovascular events after acute myocardial infarction. *N Engl. J. Med.* 354, 1578-1588. 10.1056/NEJMoa055227 16531614

[DMM052802C7] Boumann, H. A., Damen, M. J., Versluis, C., Heck, A. J., de Kruijff, B. and de Kroon, A. I. (2003). The two biosynthetic routes leading to phosphatidylcholine in yeast produce different sets of molecular species. Evidence for lipid remodeling. *Biochemistry* 42, 3054-3059. 10.1021/bi026801r 12627972

[DMM052802C8] Boumann, H. A., Chin, P. T., Heck, A. J., De Kruijff, B. and De Kroon, A. I. (2004). The yeast phospholipid N-methyltransferases catalyzing the synthesis of phosphatidylcholine preferentially convert di-C16:1 substrates both in vivo and in vitro. *J. Biol. Chem.* 279, 40314-40319. 10.1074/jbc.M406517200 15258140

[DMM052802C9] Burke, A. P., Fonseca, V., Kolodgie, F., Zieske, A., Fink, L. and Virmani, R. (2002). Increased serum homocysteine and sudden death resulting from coronary atherosclerosis with fibrous plaques. *Arterioscler. Thromb. Vasc. Biol.* 22, 1936-1941. 10.1161/01.ATV.0000035405.16217.86 12426228

[DMM052802C10] Cacciapuoti, F. (2013). Hyper-homocysteinemia: a novel risk factor or a powerful marker for cardiovascular diseases? Pathogenetic and therapeutical uncertainties. *J. Thromb. Thrombolysis* 32, 82-88. 10.1007/s11239-011-0550-4 21234645

[DMM052802C11] Cakouros, D., Mills, K., Denton, D., Paterson, A., Daish, T. and Kumar, S. (2008). dLKR/SDH regulates hormone-mediated histone arginine methylation and transcription of cell death genes. *J. Cell Biol.* 182, 481-495. 10.1083/jcb.200712169 18695041 PMC2500134

[DMM052802C12] Caudill, M. A., Wang, J. C., Melnyk, S., Pogribny, I. P., Jernigan, S., Collins, M. D., Santos-Guzman, J., Swendseid, M. E., Cogger, E. A. and James, S. J. (2001). Intracellular S-adenosylhomocysteine concentrations predict global DNA hypomethylation in tissues of methyl-deficient cystathionine beta-synthase heterozygous mice. *J. Nutr.* 131, 2811-2818. 10.1093/jn/131.11.2811 11694601

[DMM052802C13] Chen, C. W. and Armstrong, S. A. (2015). Targeting DOT1L and HOX gene expression in MLL-rearranged leukemia and beyond. *Exp. Hematol.* 43, 673-684. 10.1016/j.exphem.2015.05.012 26118503 PMC4540610

[DMM052802C14] Chintapalli, V. R., Wang, J. and Dow, J. A. (2007). Using FlyAtlas to identify better Drosophila melanogaster models of human disease. *Nat. Genet.* 39, 715-720. 10.1038/ng2049 17534367

[DMM052802C15] de Sousa, S. M. K., Krishnan, K. S. and Kenkare, U. W. (1988). Phospholipid methyltransferase from Drosophila melanogaster: purification and properties. *Insect Biochem.* 18, 377-388. 10.1016/0020-1790(88)90053-4

[DMM052802C16] Del Rizzo, P. A. and Trievel, R. C. (2011). Substrate and product specificities of SET domain methyltransferases. *Epigenetics* 6, 1059-1067. 10.4161/epi.6.9.16069 21847010 PMC3225744

[DMM052802C17] Durdevic, Z., Hanna, K., Gold, B., Pollex, T., Cherry, S., Lyko, F. and Schaefer, M. (2013). Efficient RNA virus control in Drosophila requires the RNA methyltransferase Dnmt2. *EMBO Rep.* 14, 269-275. 10.1038/embor.2013.3 23370384 PMC3589093

[DMM052802C18] Elmore, C. L., Wu, X., Leclerc, D., Watson, E. D., Bottiglieri, T., Krupenko, N. I., Krupenko, S. A., Cross, J. C., Rozen, R., Gravel, R. A. et al. (2007). Metabolic derangement of methionine and folate metabolism in mice deficient in methionine synthase reductase. *Mol. Genet. Metab.* 91, 85-97. 10.1016/j.ymgme.2007.02.001 17369066 PMC1973089

[DMM052802C19] Esse, R., Florindo, C., Imbard, A., Rocha, M. S., de Vriese, A. S., Smulders, Y. M., Teerlink, T., de Almeida, T., Castro, I., Blom, R. et al. (2013). Global protein and histone arginine methylation are affected in a tissue-specific manner in a rat model of diet-induced hyperhomocysteinemia. *Biochim. Biophys. Acta* 1832, 1708-1714. 10.1016/j.bbadis.2013.05.013 23707560

[DMM052802C20] Esse, R., Barroso, M., Tavares de Almeida, I. and Castro, R. (2019). The contribution of homocysteine metabolism disruption to endothelial dysfunction: state-of-the-art. *Int. J. Mol. Sci.* 20, 867. 10.3390/ijms20040867 30781581 PMC6412520

[DMM052802C21] Fang, J., Feng, Q., Ketel, C. S., Wang, H., Cao, R., Xia, L., Erdjument-Bromage, H., Tempst, P., Simon, J. A. and Zhang, Y. (2002). Purification and functional characterization of SET8, a nucleosomal histone H4-lysine 20-specific methyltransferase. *Curr. Biol.* 12, 1086-1099. 10.1016/S0960-9822(02)00924-7 12121615

[DMM052802C22] Fournier, P., Fourcade, J., Roncalli, J., Salvayre, R., Galinier, M. and Causse, E. (2015). Homocysteine in chronic heart failure. *Clin. Lab.* 61, 1137-1145. 10.7754/Clin.Lab.2015.141238 26554232

[DMM052802C23] Fukumoto, K., Ito, K., Saer, B., Taylor, G., Ye, S., Yamano, M., Toriba, Y., Hayes, A., Okamura, H. and Fustin, J. M. (2022). Excess S-adenosylmethionine inhibits methylation via catabolism to adenine. *Commun. Biol.* 5, 313. 10.1038/s42003-022-03280-5 35383287 PMC8983724

[DMM052802C24] Gaynor, P. M. and Carman, G. M. (1990). Phosphatidylethanolamine methyltransferase and phospholipid methyltransferase activities from Saccharomyces cerevisiae. Enzymological and kinetic properties. *Biochim. Biophys. Acta* 1045, 156-163. 10.1016/0005-2760(90)90145-N 2198947

[DMM052802C25] Gellekink, H., van Oppenraaij-Emmerzaal, D., van Rooij, A., Struys, E. A., den Heijer, M. and Blom, H. J. (2005). Stable-isotope dilution liquid chromatography-electrospray injection tandem mass spectrometry method for fast, selective measurement of S-adenosylmethionine and S-adenosylhomocysteine in plasma. *Clin. Chem.* 51, 1487-1492. 10.1373/clinchem.2004.046995 15919880

[DMM052802C26] Green, T. J., Skeaff, C. M., McMahon, J. A., Venn, B. J., Williams, S. M., Devlin, A. M. and Innis, S. M. (2010). Homocysteine-lowering vitamins do not lower plasma S-adenosylhomocysteine in older people with elevated homocysteine concentrations. *Br. J. Nutr.* 103, 1629-1634. 10.1017/S0007114509993552 20089204

[DMM052802C27] Haas, J., Frese, K. S., Park, Y. J., Keller, A., Vogel, B., Lindroth, A. M., Weichenhan, D., Franke, J., Fischer, S., Bauer, A. et al. (2013). Alterations in cardiac DNA methylation in human dilated cardiomyopathy. *EMBO Mol. Med.* 5, 413-429. 10.1002/emmm.201201553 23341106 PMC3598081

[DMM052802C28] Habisch, H., Zhang, F., Zhou, Q. and Madl, T. (2021). Exploring the arginine methylome by nuclear magnetic resonance spectroscopy. *J. Vis. Exp.* 10.3791/63245 34978288

[DMM052802C29] Jambhekar, A., Dhall, A. and Shi, Y. (2019). Roles and regulation of histone methylation in animal development. *Nat. Rev. Mol. Cell Biol.* 20, 625-641. 10.1038/s41580-019-0151-1 31267065 PMC6774358

[DMM052802C30] Jenkins, V. K., Larkin, A., Thurmond, J. and Consortium, T. F. (2022). Using FlyBase: a database of drosophila genes and genetics. In *Drosophila. Methods in Molecular Biology* (ed. C. Dahmann), pp 1-34. Vol. 2540. New York, NY: Humana. 10.1007/978-1-0716-2541-5_1 35980571

[DMM052802C31] Jin, N., Huang, L., Hong, J., Zhao, X., Chen, Y., Hu, J., Cong, X., Xie, Y. and Pu, J. (2021). Elevated homocysteine levels in patients with heart failure: A systematic review and meta-analysis. *Medicine* 100, e26875. 10.1097/MD.0000000000026875 34414939 PMC8376397

[DMM052802C32] Kerins, D. M., Koury, M. J., Capdevila, A., Rana, S. and Wagner, C. (2001). Plasma S-adenosylhomocysteine is a more sensitive indicator of cardiovascular disease than plasma homocysteine. *Am. J. Clin. Nutr.* 74, 723-729. 10.1093/ajcn/74.6.723 11722952

[DMM052802C33] Kimura, S., Sawatsubashi, S., Ito, S., Kouzmenko, A., Suzuki, E., Zhao, Y., Yamagata, K., Tanabe, M., Ueda, T., Fujiyama, S. et al. (2008). Drosophila arginine methyltransferase 1 (DART1) is an ecdysone receptor co-repressor. *Biochem. Biophys. Res. Commun.* 371, 889-893. 10.1016/j.bbrc.2008.05.003 18468516

[DMM052802C34] Kryukov, G. V., Wilson, F. H., Ruth, J. R., Paulk, J., Tsherniak, A., Marlow, S. E., Vazquez, F., Weir, B. A., Fitzgerald, M. E., Tanaka, M. et al. (2016). MTAP deletion confers enhanced dependency on the PRMT5 arginine methyltransferase in cancer cells. *Science* 351, 1214-1218. 10.1126/science.aad5214 26912360 PMC4997612

[DMM052802C35] Lefkowitz, R. J. and Willerson, J. T. (2001). Prospects for cardiovascular research. *JAMA* 285, 581-587. 10.1001/jama.285.5.581 11176863

[DMM052802C36] Lin, M. J., Tang, L. Y., Reddy, M. N. and Shen, C. K. (2005). DNA methyltransferase gene dDnmt2 and longevity of Drosophila. *J. Biol. Chem.* 280, 861-864. 10.1074/jbc.C400477200 15533947

[DMM052802C37] Lonn, E., Yusuf, S., Arnold, M. J., Sheridan, P., Pogue, J., Micks, M., McQueen, M. J., Probstfield, J., Fodor, G., Held, C. Jr et al. and Heart Outcomes Prevention Evaluation (HOPE) 2 Investigators. (2006). Homocysteine lowering with folic acid and B vitamins in vascular disease. *N Engl. J. Med.* 354, 1567-1577 10.1056/NEJMoa060900 16531613

[DMM052802C38] Loscalzo, J. (2006). Homocysteine trials--clear outcomes for complex reasons. *N Engl. J. Med.* 354, 1629-1632. 10.1056/NEJMe068060 16531615

[DMM052802C39] Malanovic, N., Streith, I., Wolinski, H., Rechberger, G., Kohlwein, S. D. and Tehlivets, O. (2008). S-adenosyl-L-homocysteine hydrolase, key enzyme of methylation metabolism, regulates phosphatidylcholine synthesis and triacylglycerol homeostasis in yeast: implications for homocysteine as a risk factor of atherosclerosis. *J. Biol. Chem.* 283, 23989-23999. 10.1074/jbc.M800830200 18591246 PMC3259781

[DMM052802C40] Mandaviya, P. R., Stolk, L. and Heil, S. G. (2014). Homocysteine and DNA methylation: a review of animal and human literature. *Mol. Genet. Metab.* 113, 243-252. 10.1016/j.ymgme.2014.10.006 25456744

[DMM052802C41] Matyash, V., Liebisch, G., Kurzchalia, T. V., Shevchenko, A. and Schwudke, D. (2008). Lipid extraction by methyl-tert-butyl ether for high-throughput lipidomics. *J. Lipid Res.* 49, 1137-1146. 10.1194/jlr.D700041-JLR200 18281723 PMC2311442

[DMM052802C42] Miller, M. W., Duhl, D. M., Winkes, B. M., Arredondo-Vega, F., Saxon, P. J., Wolff, G. L., Epstein, C. J., Hershfield, M. S. and Barsh, G. S. (1994). The mouse lethal nonagouti (a(x)) mutation deletes the S-adenosylhomocysteine hydrolase (Ahcy) gene. *EMBO J.* 13, 1806-1816. 10.1002/j.1460-2075.1994.tb06449.x 8168479 PMC395020

[DMM052802C43] Minakhina, S. and Steward, R. (2006). Melanotic mutants in Drosophila: pathways and phenotypes. *Genetics* 174, 253-263. 10.1534/genetics.106.061978 16816412 PMC1569781

[DMM052802C44] Morita, H., Seidman, J. and Seidman, C. E. (2005). Genetic causes of human heart failure. *J. Clin. Invest.* 115, 518-526. 10.1172/JCI24351 15765133 PMC1052010

[DMM052802C45] Muka, T., Koromani, F., Portilla, E., O'Connor, A., Bramer, W. M., Troup, J., Chowdhury, R., Dehghan, A. and Franco, O. H. (2016). The role of epigenetic modifications in cardiovascular disease: a systematic review. *Int. J. Cardiol.* 212, 174-183. 10.1016/j.ijcard.2016.03.062 27038728

[DMM052802C46] Nasir, K., Tsai, M., Rosen, B. D., Fernandes, V., Bluemke, D. A., Folsom, A. R. and Lima, J. A. (2007). Elevated homocysteine is associated with reduced regional left ventricular function: the multi-ethnic study of atherosclerosis. *Circulation* 115, 180-187. 10.1161/CIRCULATIONAHA.106.633750 17200444

[DMM052802C47] Noga, A. A., Stead, L. M., Zhao, Y., Brosnan, M. E., Brosnan, J. T. and Vance, D. E. (2003). Plasma homocysteine is regulated by phospholipid methylation. *J. Biol. Chem.* 278, 5952-5955. 10.1074/jbc.M212194200 12482759

[DMM052802C48] Nygard, O., Nordrehaug, J. E., Refsum, H., Ueland, P. M., Farstad, M. and Vollset, S. E. (1997). Plasma homocysteine levels and mortality in patients with coronary artery disease. *N Engl. J. Med.* 337, 230-236. 10.1056/NEJM199707243370403 9227928

[DMM052802C49] Paco, A., de Bessa Garcia, S. A. and Freitas, R. (2020). Methylation in HOX clusters and its applications in cancer therapy. *Cells* 9, 1613. 10.3390/cells9071613 32635388 PMC7408435

[DMM052802C50] Parkhitko, A. A., Binari, R., Zhang, N., Asara, J. M., Demontis, F. and Perrimon, N. (2016). Tissue-specific down-regulation of S-adenosyl-homocysteine via suppression of dAhcyL1/dAhcyL2 extends health span and life span in Drosophila. *Genes Dev.* 30, 1409-1422. 10.1101/gad.282277.116 27313316 PMC4926864

[DMM052802C51] Petrossian, T. C. and Clarke, S. G. (2011). Uncovering the human methyltransferasome. *Mol. Cell. Proteomics* 10, M110.000976. 10.1074/mcp.M110.000976 PMC3013446 20930037

[DMM052802C52] Ponrathnam, T., Saini, R., Banu, S. and Mishra, R. K. (2021). Drosophila Hox genes induce melanized pseudo-tumors when misexpressed in hemocytes. *Sci. Rep.* 11, 1838. 10.1038/s41598-021-81472-5 33469139 PMC7815749

[DMM052802C53] Qian, K., Hu, H., Xu, H. and Zheng, Y. G. (2018). Detection of PRMT1 inhibitors with stopped flow fluorescence. *Signal Transduct Target Ther.* 3, 6. 10.1038/s41392-018-0009-6 29535867 PMC5843908

[DMM052802C54] Refsum, H., Ueland, P. M., Nygard, O. and Vollset, S. E. (1998). Homocysteine and cardiovascular disease. *Ann. Rev. Med.* 49, 31-62. 10.1146/annurev.med.49.1.31 9509248

[DMM052802C55] Schaefer, M., Pollex, T., Hanna, K., Tuorto, F., Meusburger, M., Helm, M. and Lyko, F. (2010). RNA methylation by Dnmt2 protects transfer RNAs against stress-induced cleavage. *Genes Dev.* 24, 1590-1595. 10.1101/gad.586710 20679393 PMC2912555

[DMM052802C56] Schmittgen, T. D. and Livak, K. J. (2008). Analyzing real-time PCR data by the comparative C(T) method. *Nat. Protoc.* 3, 1101-1108. 10.1038/nprot.2008.73 18546601

[DMM052802C57] Shanower, G. A., Muller, M., Blanton, J. L., Honti, V., Gyurkovics, H. and Schedl, P. (2005). Characterization of the grappa gene, the Drosophila histone H3 lysine 79 methyltransferase. *Genetics* 169, 173-184. 10.1534/genetics.104.033191 15371351 PMC1448877

[DMM052802C58] Skeete, J. and DiPette, D. J. (2017). Relationship between homocysteine and hypertension: new data add to the debate. *J. Clin. Hypertens* 19, 1171-1172. 10.1111/jch.13073 PMC8030797 28942602

[DMM052802C59] Stulemeijer, I. J., De Vos, D., van Harten, K., Joshi, O. K., Blomberg, O., van Welsem, T., Terweij, M., Vlaming, H., de Graaf, E. L., Altelaar, A. F. et al. (2015). Dot1 histone methyltransferases share a distributive mechanism but have highly diverged catalytic properties. *Sci. Rep.* 5, 9824. 10.1038/srep09824 25965993 PMC4650758

[DMM052802C60] Tehlivets, O., Hasslacher, M. and Kohlwein, S. D. (2004). S-adenosyl-L-homocysteine hydrolase in yeast: key enzyme of methylation metabolism and coordinated regulation with phospholipid synthesis. *FEBS Lett.* 577, 501-506. 10.1016/j.febslet.2004.10.057 15556636

[DMM052802C61] Tehlivets, O., Malanovic, N., Visram, M., Pavkov-Keller, T. and Keller, W. (2013). S-adenosyl-L-homocysteine hydrolase and methylation disorders: yeast as a model system. *Biochim. Biophys. Acta* 1832, 204-215. 10.1016/j.bbadis.2012.09.007 23017368 PMC3787734

[DMM052802C62] Tehlivets, O., Almer, G., Brunner, M. S., Lechleitner, M., Sommer, G., Kolb, D., Leitinger, G., Diwoky, C., Wolinski, H., Habisch, H. et al. (2024). Homocysteine contributes to atherogenic transformation of the aorta in rabbits in the absence of hypercholesterolemia. *Biomed. Pharmacother.* 178, 117244. 10.1016/j.biopha.2024.117244 39116783

[DMM052802C63] Visram, M., Radulovic, M., Steiner, S., Malanovic, N., Eichmann, T. O., Wolinski, H., Rechberger, G. N. and Tehlivets, O. (2018). Homocysteine regulates fatty acid and lipid metabolism in yeast. *J. Biol. Chem.* 293, 5544-5555. 10.1074/jbc.M117.809236 29414770 PMC5900771

[DMM052802C64] Vollset, S. E., Refsum, H., Tverdal, A., Nygard, O., Nordrehaug, J. E., Tell, G. S. and Ueland, P. M. (2001). Plasma total homocysteine and cardiovascular and noncardiovascular mortality: the Hordaland Homocysteine Study. *Am. J. Clin. Nutr.* 74, 130-136. 10.1093/ajcn/74.1.130 11451728

[DMM052802C65] Wang, Y. C. and Li, C. (2012). Evolutionarily conserved protein arginine methyltransferases in non-mammalian animal systems. *FEBS J.* 279, 932-945. 10.1111/j.1742-4658.2012.08490.x 22251447

[DMM052802C66] Wolinski, H. and Kohlwein, S. D. (2015). Microscopic and spectroscopic techniques to investigate lipid droplet formation and turnover in yeast. *Method. Mol. Biol.* 1270, 289-305. 10.1007/978-1-4939-2309-0_21 25702125

[DMM052802C67] Yao, Y., Chen, P., Diao, J., Cheng, G., Deng, L., Anglin, J. L., Prasad, B. V. and Song, Y. (2011). Selective inhibitors of histone methyltransferase DOT1L: design, synthesis, and crystallographic studies. *J. Am. Chem. Soc.* 133, 16746-16749. 10.1021/ja206312b 21936531 PMC3492951

[DMM052802C68] Ye, C. and Tu, B. P. (2018). Sink into the epigenome: histones as repositories that influence cellular metabolism. *Trends Endocrinol. Metab.* 29, 626-637. 10.1016/j.tem.2018.06.002 30001904 PMC6109460

[DMM052802C69] Ye, C., Sutter, B. M., Wang, Y., Kuang, Z. and Tu, B. P. (2017). A metabolic function for phospholipid and histone methylation. *Mol. Cell* 66, −180-–193.e188. 10.1016/j.molcel.2017.02.026 PMC5482412 28366644

[DMM052802C70] Ye, C., Sutter, B. M., Wang, Y., Kuang, Z., Zhao, X., Yu, Y. and Tu, B. P. (2019). Demethylation of the protein phosphatase PP2A promotes demethylation of histones to enable their function as a methyl group sink. *Mol. Cell* 73, 1115-1126.e1116. 10.1016/j.molcel.2019.01.012 30772176 PMC6628921

[DMM052802C71] Zhang, Z., Gu, X., Fang, X., Tang, Z., Guan, S., Liu, H., Wu, X., Wang, C. and Zhao, Y. (2020). Homocysteine and the risk of cardiovascular events and all-cause death in elderly population: a community-based prospective cohort study. *Ther Clin. Risk Manag.* 16, 471-481. 10.2147/TCRM.S239496 32547044 PMC7250705

[DMM052802C72] Zhang, F., Kerbl-Knapp, J., Rodriguez Colman, M. J., Meinitzer, A., Macher, T., Vujic, N., Fasching, S., Jany-Luig, E., Korbelius, M., Kuentzel, K. B. et al. (2021). Global analysis of protein arginine methylation. *Cell Rep. Methods* 1, 100016. 10.1016/j.crmeth.2021.100016 35475236 PMC9017121

[DMM052802C73] Zhou, W., Wang, C., Chang, J., Huang, Y., Xue, Q., Miao, C. and Wu, P. (2021). RNA methylations in cardiovascular diseases, molecular structure, biological functions and regulatory roles in cardiovascular diseases. *Front. Pharmacol.* 12, 722728. 10.3389/fphar.2021.722728 34489709 PMC8417252

[DMM052802C74] Zylberstein, D. E., Bengtsson, C., Bjorkelund, C., Landaas, S., Sundh, V., Thelle, D. and Lissner, L. (2004). Serum homocysteine in relation to mortality and morbidity from coronary heart disease: a 24-year follow-up of the population study of women in Gothenburg. *Circulation* 109, 601-606. 10.1161/01.CIR.0000112581.96154.EA 14769681

