# Supplementary material for: Homocysteine and S-adenosyl-L-homocysteine impair development and methylation in yeast and flies
Source: Dis Model Mech. 2026 Jun 1;19(5):dmm052802. doi: 10.1242/dmm.052802 (PMC13312927; doi:10.1242/dmm.052802)

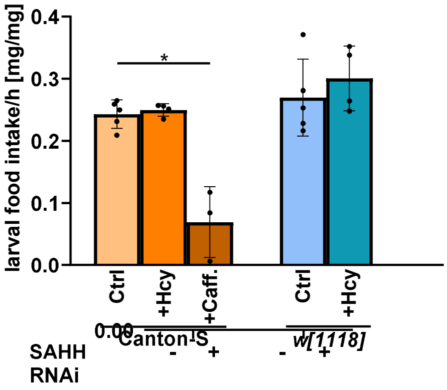

Fig. S1. Food intake in Canton-S and *w[1118] Drosophila* larvae on standard or Hcy-supplemented food. Food intake in Canton-S and *w[1118]* non-migratory L3 larvae on standard-, Hcy-supplemented (20 mM)- or caffeine (positive control for aversive cues; 0.2% w/v)-supplemented food per hour normalized to larval weight (n=3–5). Canton-S statistics were calculated with Students *t*-tests pairwise comparisons with Games-Howell correction for multiple testing. *w[1118]* statistic was calculated with Students *t*-test (* = p ≤ 0.05, ** = p ≤ 0.01, *** = p ≤ 0.001). All statistics are shown in Supplemental Data 3.

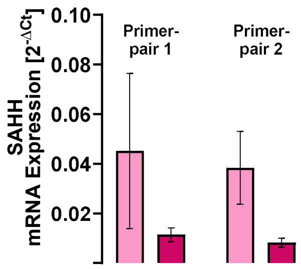

Fig. S2. SAHH mRNA expression in *Drosophila* larvae ubiquitously expressing a mCherry RNAi transgene or a SAHH RNAi transgene. Analysis of SAHH mRNA expression in migratory L3 *Drosophila* larvae ubiquitously expressing a mCherry RNAi transgene (control) or a SAHH RNAi transgene via RT-PCR using two different sets of SAHH primers (n = 2). Primer-pair 1 = exon spanning SAHH primer pair, Primer-pair 2 = primer-pair amplifying both SAHH isoforms.

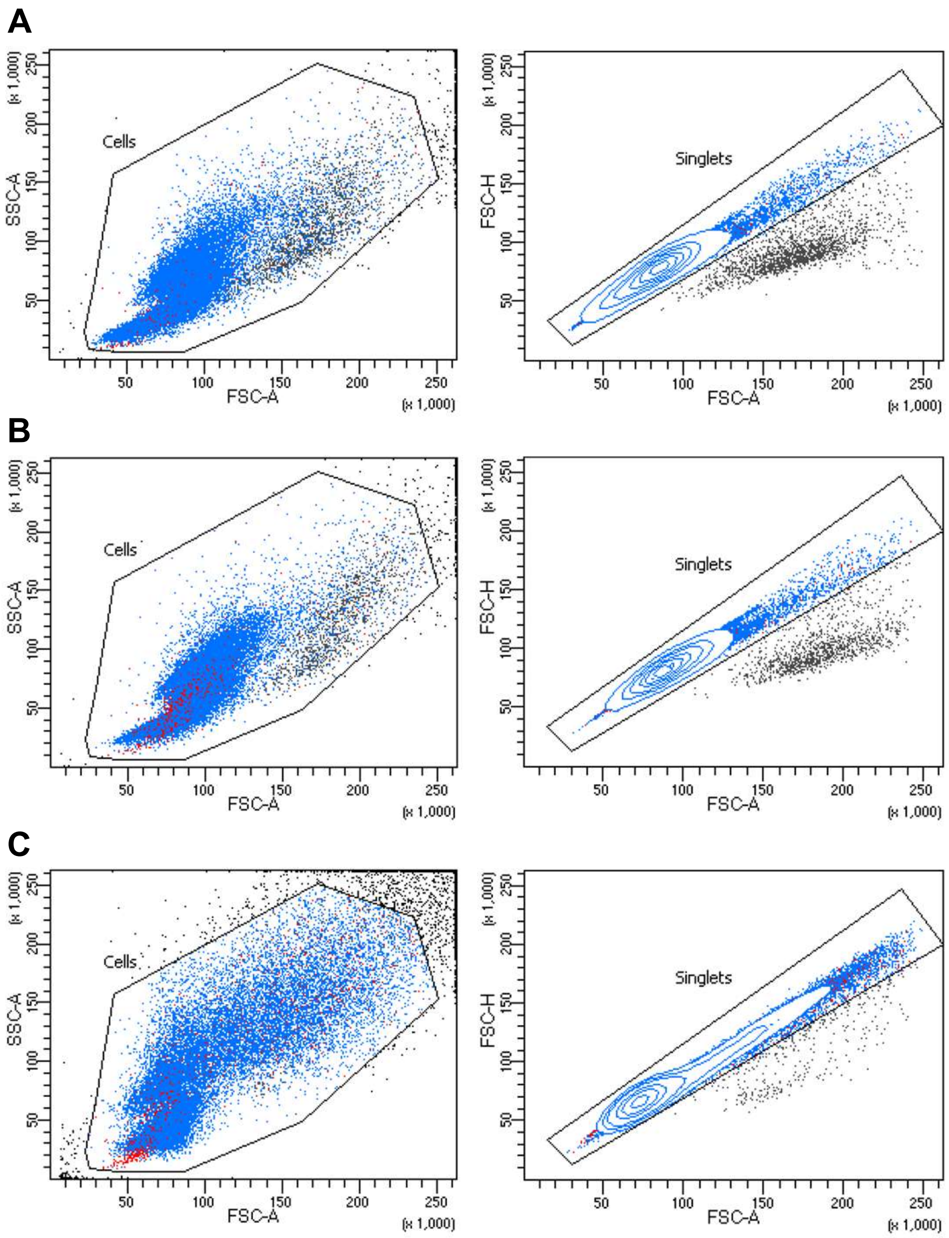

Fig. S3. Representative light scatter patterns of flow cytometry analysis in yeast supplementation and genetic models. Patterns of forward scatter area (FSC-A) versus side scatter area (SSC-A; left) and patterns of FSC-A versus front scatter height (FSC-H; right) of wild type yeast grown in the absence (A) or presence (B) of 5 mM Hcy and in the yeast Δ*sah1* mutant grown without Hcy supplementation (C). Black encircled areas represent gating for primary cells (left) as well as single-cell gating (right). Round blue areas show regions with very high density of detected signals. Per sample 30,000 events were recorded. Blue spots represent single cells within boundaries of both gates, black spots represent cells discriminated as cell debris or doublets, red spots represent dead cells positively screened for propidium iodide fluorescence signal.

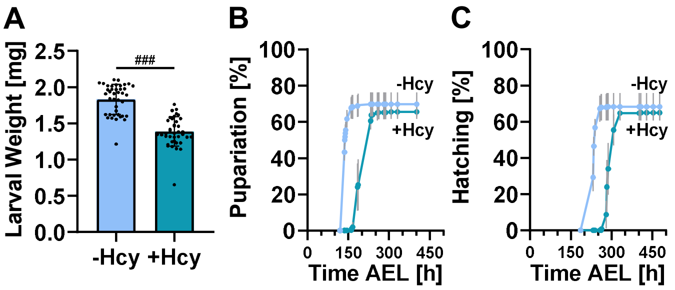

Fig. S4. Developmental delay in *w[1118] Drosophila* grown on Hcy-supplemented food. Migratory L3 larval weight (n=40) (A), pupariation (n=4) (B) and adult hatching rates (n=4) (C) of *w[1118] Drosophila* larvae and flies grown on standard fly food or food containing 20 mM Hcy . For larval weight, statistic was calculated with Mann-Whitney-U pairwise comparison (# = p ≤ 0.05, ## = p ≤ 0.01, ### = p ≤ 0.001). All statistics are shown in Supplemental Data 3.

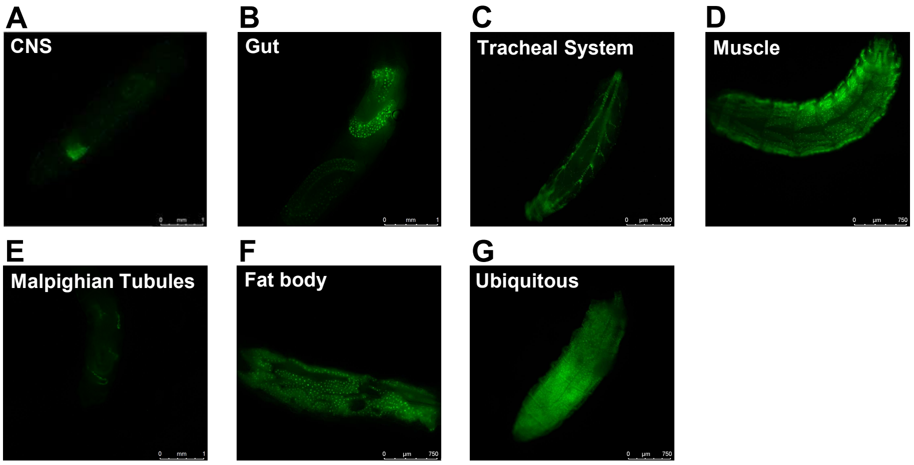

Fig. S5. Confirmation of organ-specificity of *in vivo* transgene in *Drosophila* larvae. Transgenic fly lines controlling organ-specific gene expression were crossed against a transgenic GFP-reporter fly line, and the resulting fluorescence was imaged at the migratory L3 larval stage. Expression of organ-specific GFP signals in *Drosophila* progeny of GFP reporter line crossed with *Drosophila* driver lines for CNS (A), gut (B), tracheal system (C), muscle (D), Malpighian tubules (E), fat body (F) or ubiquitous (G) expression. Potential off-target GFP expression below detection limit cannot be excluded.

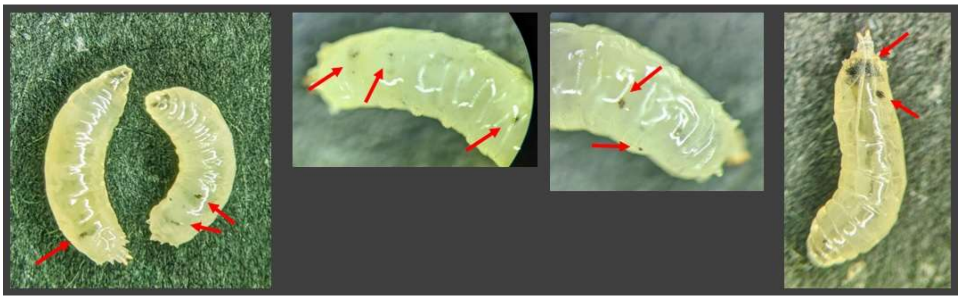

Fig. S6. Putative melanotic tumors in *Drosophila* larvae globally expressing SAHH RNAi. Photographs of 9 days old Canton-S *Drosophila* larvae ubiquitously expressing SAHH RNAi grown on standard food. Red arrows point to putative melanotic tumors.

Table S1. Yeast strains used in this study

Strain	Genotype	Source
**WT**	*MAT a his3Δ1 leu2Δ0 ura3Δ0*	Visram et al. (2018)
Δsah1	*MAT a his3Δ1 leu2Δ0 ura3Δ0 sah1::kanMX4*	Visram et al. (2018)

Table S2. *Drosophila* fly lines used in this study

Trivial Name	Function	Genotype	Internal Stock No.	Source	Source Stock No.
**Canton-S**	wild type control stock	-	RKF 1679	-	BDSC* 64349
w[1118]	white mutant control stock	w^{1118}; +/+; +/+;	RKF 1084	-	VDRC** 6000
**Ubiquitous**	Ubiquitous driver	*w*; +/+; P{GAL4-da.G32}*	RKF 908	Wodarz et al. (1995)	-
**RNAi ctrl**	mCherry RNAi effector	*y[1] sc* v[1]; P{y[+t7.7] v[+t1.8]=VALIUM20-mCherry}attP2*	RKF 1645	-	BDSC 35785
**SAHH RNAi**	SAH hydrolase RNAi effector	*w[1118] y[1] float. sc* float. v[1] sev[21] float. ; P{y[+t7.7] v[+t1.8]=TriP.HMS05799}attP40;*	RKF 2416	This work based on BDSC*	based on BDSC 67848
**GFP**	GFP reporter	*+; P{w[+mC]=UAS-Stinger}2; +/+*	RKF 1171	Barolo et al. (2000)	-
**CNS**	Central nervous system-specific driver	*w*; +/+; P{w[+mW.hs]=GAL4-Nrv2-3} P{w[+m*]UAS-GFP}*	RKF 211	Sun et al. (1999)	-
**Gut**	Gut enterocyte-specific driver	*w[1118]; P{w[+mC]=mex1-GAL4.2.1}10-8*	CHF 2396	-	BDSC 91368
**Tracheal System**	Tracheal system-specific driver	*w*; P{UAS-GFP::nLacZ} / CyO float; P{btl-GAL4} / TM3 Sb* Ser**	RKF 1912	-	KSC*** 109132
**Muscle**	Muscle-specific driver	*mef2-Gal4*	IPF 2174	-	BDSC 27390
**Malpighian Tubules**	Malpighian tubules-specific driver	*+/+; UO-Gal4/CyO float; +/+*	JRF 1254	Terhzaz et al. (2010)	-
**Fat body**	Fat body-specific driver	*w*; +/+; P{Lpp-GAL4.B}/TM3, P{w[+mC]=ActGFP}JMR2, Ser[1]*	RKF 1582	This work; based on Brankatschk and Eaton (2010)	-

* = Bloomington Drosophila Stock Centre, ** = Vienna Drosophila Resource Center, *** = KYOTO Stock Center

Table S3. *Drosophila* RT-qPCR primers

Target	Forward primer sequence	Reverse primer sequence	Source
SAHH (exon spanning)	5’- AGT ACG GCC CAT CTA AGC C - 3'	5' - CGG CAG CAT TAT CCT GGG T - 3'	Fly RNAi Database, identifier PP25254
SAHH (both isoforms)	5' - AGC CCC TGA ACA TGA TCC TG - 3'	5' - CGA CCC TCC TTG AAC ATC TTG T - 3'	Fly RNAi Database, identifier PP36991
GAPDH	5' - GTC GGG CTT GTA GGC ATC C - 3'	5' - AGG CAT CCA CTC ACT TGA AGG - 3'	Beaucher et al. (2007)

Disease Models & Mechanisms • Supplementary information

Dataset 1. Chemicals and consumables

Name	Company	Product No.
Difco yeast nitrogen base	Becton, Dickinson and Company	233520
ammonium sulfate	Roth	3746.1
glucose	neoFroxx	LC-5042.2
complete supplement mixture (CSM)	mp biomedicals	4500022
DL-Homocysteine (Hcy)	Sigma Aldrich	H4628
baker's yeast	Gewürzmühle Brecht	03462
soy flour	Soja Austria	Sojamehl
agar-agar	Gewürzmühle Brecht	00162
maize flour	Haindl Mühle	Maismehl
beet syrup	Grafschafter	01936
malt	CSM Austria	728985
propionic acid	Merck	P5561
methyl-4-hydrobenzoate (nipagin)	Merck	W271004
water, MS-grade	Chem-Lab	CL02.0240.2500
agar	Fluka Analytical	05038
apple juice	Spar Sbudget	2020004602114
sugar	Spar Sbudget	2020000710103
ethanol (EtOH)	Chem-Lab	CL00.0529.2500
brilliant blue	funfood4you.com	-
caffeine	Sigma Aldrich	C0750
methanol (MeOH), MS-grade	Honeywell|Riedel-de Haen	34966
ammonium acetate	J.T.Baker	0599-08
formic acid	Merck	Z0536902 849
S-adenosylhomocysteine-d4 (SAH-d4)	CDN Isotopes	D-6452
S-adenosylmethionine-d3 (SAM-d3)	CDN Isotopes	D-4093
S-adenosylhomocysteine (SAH)	Sigma Aldrich	A9384
S-adenosylmethionine (SAM)	Sigma Aldrich	A2408
sodium hydroxide (NaOH), 1 N	Roth	K021.1
β-mercaptoethanol	Sigma Aldrich	M6250
tri-chloroacetic acid (TCA)	Roth	8789.2
Tris	Roth	4855.2
sodium dodecyl sulfate (SDS), pellets	Roth	CN30.3
glycerol	Merck	1.04092.1000
bromophenol blue	Merck	1.11746.0005
dithiotreitol (DTT)	Sigma Aldrich	DTT-RO
phosphate buffered saline (PBS), pH 7.4, 1x	gibco	10010-015
Triton X-100	Bio-Rad	1610407
phenylmethylsulfonyl fluoride (PMSF)	Sigma Aldrich	P7626
hydrochloric acid (HCl), 1 N	Roth	K025.1
skim milk	Roth	T145.3
Tween-20	Merck	P9416
methyl-tert-butyl ether (MTBE)	Promochem	SO-5398-B025
phosphatidylcholine 38:0 (PC 38:0)	Avanti Polar Lipids Inc.	855676P
phosphatidylethanolamine 34:0 (PE 34:0)	Avanti Polar Lipids Inc.	830756P
lysophosphatidylcholine 17:0 (LPC 17:0)	Avanti Polar Lipids Inc.	855676P
lysophosphatidylethanolamine 17:1 (LPE 17:1)	Avanti Polar Lipids Inc.	856707C
isopropanol	Chem-Lab	CL00.0926.2500
phosphoric acid	Roth	9079.1
disodium phosphate (Na_2HPO_4)	VWR	80731-078
3-trimethylsilyl propionic acid-2,2,3,3-d4 sodium salt (TSP)	Alfa Aesar	A1448
deuterium oxide (D_2O)	Cambridge Isotope Laboratories	DLM-6-1000
sodium hydroxide (NaOH), 33 %	VWR	BDH7363-4
hydrochloric acid (HCl), 32 %	Merck	1.00313.2500
propidium iodide (PI)	Applichem	A2261.0100

List of Consumables

Name	Company	Product No.
Drosophila Containers, 68 mL	Greiner bio one	217101
mite-tight stoppers, ø 36 mm, 30 mm height	K-TK	1002S
petri dishes, 60 x 15 mm with vents	Greiner bio one	628102
petri dishes, 94 x 16 mm with vents	Greiner bio one	633180
2 mL safe-seal tubes	Sarstedt	72.695.500
steel ball, ø 5 mm	Askubal	504942
glass beads SiLibeads Typ S, ø 0.4 - 0.6 mm	SiLi Sigmund Lindner	45015FL
Bond Elut PBA columns	Agilent	12102019
BEH C18 column, 3.0 x 150 mm, ø 1.7 µm	Waters	186004690
RNeasy Minikit	Quiagen	74104
DNAse I	New England Biolabs	M0303S
DNAse I reaction buffer	New England Biolabs	B0303S
Invitrogen superscript III One-Step RT-PCR System	Thermo Fisher Scientific	12574018
iTaq Universal SYBR Green Supermix	Bio-Rad	1725121
Invitrogen NuPAGE, 12 % BT 1.0, 15-well	Thermo Fisher Scientific	NP0343BOX
Immobilon-P Transfer Membrane PVDF	Merck	IPVH00010
GAPDH antibody	Cell Signaling Technology	2118S
MMA antibody	Cell Signaling Technology	8711
H3 antibody	Abcam	ab1791
H3K4Me3 antibody	Abcam	ab8580
H3K36Me2 antibody	Abcam	ab9049
H3K36Me3 antibody	Abcam	ab9050
H3K79Me1 antibody	Fred van Leeuwen lab	-
H3K79Me2 antibody	Fred van Leeuwen lab	-
H3K79Me3 antibody	Fred van Leeuwen lab	-
secondary α-rabbit horseradish peroxidase-conjugated antibody	Sigma Aldrich	A0545
Clarity Western ECL Substrate Kit	Bio-Rad	170-5061
Precellys tubes & Zirconium oxide beads, ø 1.4 mm	Bertin Technologies	P000927-LYSK0-A
honeycomb plates HC2	Bioscreen	95025BIO
microplate 96-well, round bottom	Greiner bio one	650101

Dataset 2. Phospholipid target mass lists

Yeast Phospholipid Target Mass-List

Lipid Class	Lipid Species	Precursor Mass [g/mol +1 H]	Product Mass [g/mol + 1 H]	Fragmentor Voltage [V]	Collision Energy [V]	Cell Accelerator [V]	Retention Time [min]
DMPE	DMPE 32_0	720.6	551.6	164	28	5	15.5
DMPE	DMPE 32_1	718.6	549.6	164	28	5	15.5
DMPE	DMPE 32_2	716.6	547.6	164	28	5	15.5
DMPE	DMPE 34_0	748.6	579.6	164	28	5	15.5
DMPE	DMPE 34_1	746.6	577.6	164	28	5	15.5
DMPE	DMPE 34_2	744.6	575.6	164	28	5	15.5
DMPE	DMPE 36_0	776.6	607.6	164	28	5	15.5
DMPE	DMPE 36_1	774.6	605.6	164	28	5	15.5
DMPE	DMPE 36_2	772.6	603.6	164	28	5	15.5
PC	IST PC 38_0	818.7	184.1	164	28	5	15.5
PE	IST PE 34_0	720.6	579.6	131	20	5	15.5
MMPE	MMPE 32_0	706.5	551.5	150	27	5	16
MMPE	MMPE 32_1	704.5	549.5	150	27	5	16
MMPE	MMPE 32_2	702.5	547.5	150	27	5	16
MMPE	MMPE 34_0	734.6	579.6	150	27	5	16
MMPE	MMPE 34_1	732.6	577.6	150	27	5	16
MMPE	MMPE 34_2	730.6	575.6	150	27	5	16
MMPE	MMPE 36_0	762.6	607.6	150	27	5	16
MMPE	MMPE 36_1	760.6	605.6	150	27	5	16
MMPE	MMPE 36_2	758.6	603.6	150	27	5	16
PC	PC 30_0	706.6	184.1	164	28	5	15.5
PC	PC 30_1	704.6	184.1	164	28	5	15.5
PC	PC 30_2	702.6	184.1	164	28	5	15.5
PC	PC 32_0	734.9	184.1	164	28	5	15.5
PC	PC 32_1	732.9	184.1	164	28	5	15.5
PC	PC 32_2	730.9	184.1	164	28	5	15.5
PC	PC 33_0	748.59	184.1	164	28	5	15.5
PC	PC 33_1	746.57	184.1	164	28	5	15.5
PC	PC 33_2	744.9	184.1	164	28	5	15.5
PC	PC 34_0	762.9	184.1	164	28	5	15.5
PC	PC 34_1	760.9	184.1	164	28	5	15.5
PC	PC 34_2	758.9	184.1	164	28	5	15.5
PC	PC 35_2	772.59	184.1	164	28	5	15.5
PC	PC 36_0	790.6	184.1	164	28	5	15.5
PC	PC 36_1	788.6	184.1	164	28	5	15.5
PC	PC 36_2	786.6	184.1	164	28	5	15.5
PE	PE 30_0	664.5	523.5	131	20	5	15.5
PE	PE 30_1	662.5	521.5	131	20	5	15.5
PE	PE 30_2	660.5	519.5	131	20	5	15.5
PE	PE 32_0	692.5	551.5	131	20	5	15.5
PE	PE 32_1	690.3	549.3	131	20	5	15.5
PE	PE 32_2	688.2	547.2	131	20	5	15.5
PE	PE 34_0	720.3	579.3	131	20	5	15.5
PE	PE 34_1	718.3	577.3	131	20	5	15.5
PE	PE 34_2	716.3	575.3	131	20	5	15.5
PE	PE 36_0	748.6	607.3	131	20	5	15.5
PE	PE 36_1	746.3	605.3	131	20	5	15.5
PE	PE 36_2	744.2	603.2	131	20	5	15.5

Drosophila Phospholipid Target Mass-List

Lipid Class	Lipid Species	Precursor Mass [g/mol +1 H]	Product Mass [g/mol + 1 H]	Fragmentor Voltage [V]	Collision Energy [V]	Cell Accelerator [V]	Retention Time [min]
PC	PC 24_0	622.4	184.1	164	28	5	11.7
PC	PC 26_0	650.5	184.1	164	28	5	12.79
PC	PC 26_1	648.5	184.1	164	28	5	12.17
PC	PC 28_0	678.5	184.1	164	28	5	13.8
PC	PC 28_1	676.5	184.1	164	28	5	13.2
PC	PC 28_2	674.5	184.1	164	28	5	12.6
PC	PC 30_0	706.5	184.1	164	28	5	14.84
PC	PC 30_1	704.5	184.1	164	28	5	14.0
PC	PC 30_2	702.5	184.1	164	28	5	13.42
PC	PC 30_3	700.5	184.1	164	28	5	12.86
PC	PC 32_0	734.6	184.1	164	28	5	15.85
PC	PC 32_1	732.6	184.1	164	28	5	15.015
PC	PC 32_2	730.5	184.1	164	28	5	14.3
PC	PC 32_3	728.5	184.1	164	28	5	13.72
PC	PC 32_4	726.5	184.1	164	28	5	13.15
PC	PC 32_5	724.5	184.1	164	28	5	12.55
PC	PC 34_0	762.6	184.1	164	28	5	16.83
PC	PC 34_1	760.6	184.1	164	28	5	16.0
PC	PC 34_2	758.6	184.1	164	28	5	15.3
PC	PC 34_3	756.6	184.1	164	28	5	14.5
PC	PC 34_4	754.5	184.1	164	28	5	13.96
PC	PC 34_5	752.5	184.1	164	28	5	13.4
PC	PC 34_6	750.5	184.1	164	28	5	12.9
PC	PC 36_0	790.6	184.1	164	28	5	17.75
PC	PC 36_1	788.6	184.1	164	28	5	16.96
PC	PC 36_2	786.6	184.1	164	28	5	16.15
PC	PC 36_3	784.6	184.1	164	28	5	15.47
PC	PC 36_4	782.6	184.1	164	28	5	14.81
PC	PC 36_5	780.6	184.1	164	28	5	14.24
PC	PC 36_6	778.5	184.1	164	28	5	13.68
PC	PC 38_1	816.6	184.1	164	28	5	17.86
PC	PC 38_2	814.6	184.1	164	28	5	17.23
PC	PC 38_3	812.6	184.1	164	28	5	16.38
PC	PC 38_4	810.6	184.1	164	28	5	15.7
PC	IST PC 38_0	818.7	184.1	164	28	5	18.5
PE	PE 26_0	608.4	467.4	131	20	5	13.05
PE	PE 28_0	636.5	495.5	131	20	5	14.09
PE	PE 28_1	634.4	493.4	131	20	5	13.45
PE	PE 28_2	632.4	491.4	131	20	5	12.9
PE	PE 30_0	664.5	523.5	131	20	5	15.11
PE	PE 30_1	662.5	521.5	131	20	5	14.27
PE	PE 30_2	660.5	519.5	131	20	5	13.73
PE	PE 32_0	692.5	551.5	131	20	5	16.13
PE	PE 32_1	690.5	549.5	131	20	5	15.28
PE	PE 32_2	688.5	547.5	131	20	5	14.58
PE	PE 32_3	686.5	545.5	131	20	5	14.04
PE	PE 34_0	720.6	579.6	131	20	5	16.26

PE	PE 34_1	718.5	577.5	131	20	5	16.26
PE	PE 34_2	716.5	575.5	131	20	5	15.58
PE	PE 34_3	714.5	573.5	131	20	5	14.765
PE	PE 34_4	712.5	571.5	131	20	5	14.21
PE	PE 34_5	710.5	569.5	131	20	5	13.68
PE	PE 36_0	748.6	607.6	131	20	5	18.01
PE	PE 36_1	746.6	605.6	131	20	5	17.2
PE	PE 36_2	744.6	603.6	131	20	5	16.38
PE	PE 36_3	742.5	601.5	131	20	5	15.735
PE	PE 36_4	740.5	599.5	131	20	5	15.09
PE	PE 36_5	738.5	597.5	131	20	5	14.5
PE	PE 36_6	736.5	595.5	131	20	5	13.95
PE	PE 38_1	774.6	633.6	131	20	5	18.085
PE	PE 38_2	772.6	631.6	131	20	5	17.47
PE	PE 38_3	770.6	629.6	131	20	5	16.65
PE	PE 38_4	768.6	627.6	131	20	5	17.23
PE	PE 38_5	766.5	625.5	131	20	5	16.41
PE	PE 38_6	764.5	623.5	131	20	5	15.74
PE	PE 38_7	762.5	621.5	131	20	5	15.09
PE	PE 40_1	802.6	661.6	131	20	5	18.91
PE	PE 40_2	800.6	659.6	131	20	5	18.3
PE	PE 40_3	798.6	657.6	131	20	5	17.55
PE	PE 40_4	796.6	655.6	131	20	5	18.08
PE	PE 40_5	794.6	653.6	131	20	5	17.425
DMPE	DMPE 30_0	692.5	523.5	164	28	5	15.05
DMPE	DMPE 30_1	690.5	521.5	164	28	5	14.19
DMPE	DMPE 32_1	718.5	549.5	164	28	5	15.22
DMPE	DMPE 32_2	716.5	547.5	164	28	5	14.48
DMPE	DMPE 32_3	714.5	545.5	164	28	5	13.91
DMPE	DMPE 34_1	746.6	577.6	164	28	5	16.18
DMPE	DMPE 34_2	744.6	575.6	164	28	5	15.53
DMPE	DMPE 34_3	742.5	573.5	164	28	5	14.69
DMPE	DMPE 34_4	740.5	571.5	164	28	5	14.14
DMPE	DMPE 36_1	774.6	605.6	164	28	5	17.18
DMPE	DMPE 36_2	772.6	603.6	164	28	5	16.22
DMPE	DMPE 36_3	770.6	601.6	164	28	5	15.67
DMPE	DMPE 36_4	768.6	599.6	164	28	5	15.035
DMPE	DMPE 36_5	766.5	597.5	164	28	5	14.4
MMPE	MMPE 30_0	678.5	523.5	150	27	5	15.11
MMPE	MMPE 30_1	676.5	521.5	150	27	5	14.35
MMPE	MMPE 32_0	706.5	551.5	150	27	5	16.055
MMPE	MMPE 32_1	704.5	549.5	150	27	5	15.26
MMPE	MMPE 32_2	702.5	547.5	150	27	5	14.61
MMPE	MMPE 32_3	700.5	545.5	150	27	5	14.09
MMPE	MMPE 34_1	732.6	577.6	150	27	5	16.245
MMPE	MMPE 34_2	730.5	575.5	150	27	5	15.58
MMPE	MMPE 34_3	728.5	573.5	150	27	5	14.725
MMPE	MMPE 34_4	726.5	571.5	150	27	5	14.165
MMPE	MMPE 36_1	760.6	605.6	150	27	5	17.16
MMPE	MMPE 36_2	758.6	603.6	150	27	5	16.33

Disease Models & Mechanisms • Supplementary information

MMPE	MMPE 36_3	756.6	601.6	150	27	5	15.7
MMPE	MMPE 36_4	754.5	599.5	150	27	5	15.055
MMPE	MMPE 36_5	752.5	597.5	150	27	5	14.5
MMPE	MMPE 38_0	790.6	635.6	150	27	5	21.16
MMPE	MMPE 38_1	788.6	633.6	150	27	5	20.69
MMPE	MMPE 38_5	780.6	625.6	150	27	5	16.5
MMPE	MMPE 38_6	778.5	623.5	150	27	5	15.67
MMPE	MMPE 38_7	776.5	621.5	150	27	5	15.1
MMPE	MMPE 40_0	818.7	663.7	150	27	5	21.9
MMPE	MMPE 40_1	816.6	661.6	150	27	5	21.3
MMPE	MMPE 40_2	814.6	659.6	150	27	5	20.95
MMPE	MMPE 42_0	846.7	691.7	150	27	5	22.29
MMPE	MMPE 42_1	844.7	689.7	150	27	5	21.9
MMPE	MMPE 42_2	842.7	687.7	150	27	5	21.43
MMPE	MMPE 42_3	840.6	685.6	150	27	5	21.02
MMPE	MMPE 44_0	874.7	719.7	150	27	5	22.9
MMPE	MMPE 44_1	872.7	717.7	150	27	5	22.49
MMPE	MMPE 44_2	870.7	715.7	150	27	5	21.99

Dataset 3. Statistics

Yeast SAH quantifications

One-way ANOVA with pairwise t-tests and Bonferroni correction for multiple testing

Sample 1 - Sample 2	adjusted p-value	significance-level
WT - +Hcy	0,002	**
WT - *sah1*	< 0,001	***
+Hcy - *sah1*	< 0,001	***

Yeast SAM quantifications

Independent samples Kruskal-Wallis test with pairwise Mann-Whitney-U tests and Bonferroni correction for multiple testing

Sample 1 - Sample 2	p-value	adjusted p-value	significance-level
WT - +Hcy	0,695	1,000	-
WT - *sah1*	0,031	0,093	-
+Hcy - *sah1*	0,011	0,032	#

Yeast SAM/SAH ratios

One-way ANOVA with pairwise t-tests and Bonferroni correction for multiple testing

Sample 1 - Sample 2	adjusted p-value	significance-level
WT - +Hcy	0,001	**
WT - *sah1*	1,000	-
+Hcy - *sah1*	< 0,001	***

Drosophila **SAH quantifications**

Independent samples t-tests

Sample 1 - Sample 2	p-value	significance-level
-Hcy - +Hcy (Canton-S)	0,002	**
-Hcy - +Hcy (*w[1118]*)	< 0,001	***
ctrl RNAi - SAHH RNAi	< 0,001	***

Drosophila **SAM quantifications**

Independent samples t-tests

Sample 1 - Sample 2	p-value	significance-level
-Hcy - +Hcy (Canton-S)	0,517	-
-Hcy - +Hcy (*w[1118]*)	0,004	**
ctrl RNAi - SAHH RNAi	< 0,001	***

Drosophila **SAM/SAH ratios**

Independent samples t-tests

Sample 1 - Sample 2	p-value	significance-level
-Hcy - +Hcy (Canton-S)	0,023	*
-Hcy - +Hcy (*w[1118]*)	0,002	**
ctrl RNAi - SAHH RNAi	0,002	**

Disease Models & Mechanisms • Supplementary information

Yeast cell death analysis

One-way ANOVA with pairwise t-tests and Bonferroni correction for multiple testing

Sample 1 - Sample 2	adjusted p-value	significance-level
WT - +Hcy	1,000	-
WT - *sah1*	0,020	*
+Hcy - *sah1*	0,051	-

Yeast cell size analysis

Independent samples Kruskal-Wallis test with pairwise Mann-Whitney-U tests and Bonferroni correction for multiple testing

Sample 1 - Sample 2	p-value	adjusted p-value	significance-level
WT - +Hcy	0,095	0,285	-
WT - *sah1*	0,006	0,018	#
+Hcy - *sah1*	0,280	0,840	-

***Drosophila* larval weight**

Independent samples Mann-Whitney-U test

Sample 1 - Sample 2	p-value	significance-level
-Hcy - +Hcy	< 0.001	###

Hatching rate in *Drosophila* with organ-specific RNAi expression

Independent samples Students t-tests or Mann-Whitney-U tests

Organ-specific expression	test	p-value	significance-level
CNS	Students t-test	0,005	**
Gut	Students t-test	0,166	-
Tracheal System	Students t-test	< 0,001	***
Muscles	Students t-test	0,007	**
Malpighian Tubules	Mann-Whitney-U	0,486	-
Fat body	Students t-test	0,647	-
Ubiquitous	Students t-test	0,023	*

Yeast relative PE/Total PL

One-way ANOVA with pairwise t-tests and Bonferroni correction for multiple testing

Sample 1 - Sample 2	adjusted p-value	significance-level
WT - +Hcy	< 0,001	***
WT - *sah1*	< 0,001	***
+Hcy - *sah1*	< 0,001	***

Yeast relative MMPE/Total PL

One-way ANOVA with pairwise t-tests and Games-Howell correction for multiple testing

Sample 1 - Sample 2	adjusted p-value	significance-level
WT - +Hcy	< 0,001	***
WT - *sah1*	0,398	-
+Hcy - *sah1*	< 0,001	***

Yeast relative DMPE/Total PL

Independent samples Kruskal-Wallis test with pairwise Mann-Whitney-U tests and Bonferroni correction for multiple testing

Sample 1 - Sample 2	p-value	adjusted p-value	significance-level
WT - +Hcy	0,113	0,340	-
WT - *sah1*	0,002	0,005	##
+Hcy - *sah1*	0,113	0,340	-

Yeast relative PC/Total PL

One-way ANOVA with pairwise t-tests and Bonferroni correction for multiple testing

Sample 1 - Sample 2	adjusted p-value	significance-level
WT - +Hcy	< 0,001	***
WT - *sah1*	< 0,001	***
+Hcy - *sah1*	< 0,001	***

Yeast PC/PE ratios

One-way ANOVA with pairwise t-tests and Games-Howell correction for multiple testing

Sample 1 - Sample 2	adjusted p-value	significance-level
WT - +Hcy	< 0,001	***
WT - *sah1*	< 0,001	***
+Hcy - *sah1*	< 0,001	***

***Drosophila* relative PE/Total PL**

Independent samples t-tests

Sample 1 - Sample 2	p-value	significance-level
-Hcy - +Hcy	0,465	-
ctrl RNAi - SAHH RNAi	0,003	**

***Drosophila* relative MMPE/Total PL**

Independent samples t-tests

Sample 1 - Sample 2	p-value	significance-level
-Hcy - +Hcy	0,013	*
ctrl RNAi - SAHH RNAi	< 0,001	***

***Drosophila* relative DMPE/Total PL**

Independent samples t-test (dietary) and independent samples Mann-Whitney-U test (genetic)

Sample 1 - Sample 2	p-value	significance-level
-Hcy - +Hcy (dietary)	0,011	*
ctrl RNAi - SAHH RNAi (genetic)	0,100	-

***Drosophila* relative PC/Total PL**

Independent samples t-tests

Sample 1 - Sample 2	p-value	significance-level
-Hcy - +Hcy	0,278	-
ctrl RNAi - SAHH RNAi	0,183	-

***Drosophila* PC/PE ratios**

Independent samples t-tests

Sample 1 - Sample 2	p-value	significance-level
-Hcy - +Hcy	0,269	-
ctrl RNAi - SAHH RNAi	< 0,001	***

Yeast total protein ADMA to total protein arginine ratios

One-way ANOVA with pairwise t-tests and Bonferroni correction for multiple testing

Sample 1 - Sample 2	adjusted p-value	significance-level
WT - +Hcy	< 0,001	***
WT - *sah1*	< 0,001	***
+Hcy - *sah1*	1,000	-

***Drosophila* total protein ADMA to total protein arginine ratios**

Independent samples Mann-Whitney-U test

Sample 1 - Sample 2	p-value	significance-level
-Hcy - +Hcy	0.151	-

Disease Models & Mechanisms • Supplementary information

Yeast H3K4Me3 relative quantification

One-way ANOVA with pairwise t-tests and Bonferroni correction for multiple testing

Sample 1 - Sample 2	adjusted p-value	significance-level
WT - +Hcy	0,020	*
WT - *sah1*	< 0,001	***
+Hcy - *sah1*	0,181	-

Yeast H3K36Me2 relative quantification

One-way ANOVA with pairwise t-tests and Bonferroni correction for multiple testing

Sample 1 - Sample 2	adjusted p-value	significance-level
WT - +Hcy	0,600	-
WT - *sah1*	< 0,001	***
+Hcy - *sah1*	0,004	**

Yeast H3K36Me3 relative quantification

One-way ANOVA with pairwise t-tests and Bonferroni correction for multiple testing

Sample 1 - Sample 2	adjusted p-value	significance-level
WT - +Hcy	0,001	**
WT - *sah1*	< 0,001	***
+Hcy - *sah1*	< 0,001	***

Yeast H3K79Me1 relative quantification

One-way ANOVA with pairwise t-tests and Bonferroni correction for multiple testing

Sample 1 - Sample 2	adjusted p-value	significance-level
WT - +Hcy	0,016	*
WT - *sah1*	0,077	-
+Hcy - *sah1*	1,000	-

Yeast H3K79Me2 relative quantification

Independent samples Kruskal-Wallis test with pairwise Mann-Whitney-U tests and Bonferroni correction for multiple testing

Sample 1 - Sample 2	p-value	adjusted p-value	significance-level
WT - +Hcy	0,624	1,000	-
WT - *sah1*	0,202	0,607	-
+Hcy - *sah1*	0,433	1,000	-

Yeast H3K79Me3 relative quantification

One-way ANOVA with pairwise t-tests and Bonferroni correction for multiple testing

Sample 1 - Sample 2	adjusted p-value	significance-level
WT - +Hcy	0,004	**
WT - *sah1*	0,139	-
+Hcy - *sah1*	0,142	-

***Drosophila* H3K4Me3 relative quantification**

Independent samples t-tests

Sample 1 - Sample 2	p-value	significance-level
-Hcy - +Hcy	0,794	-
ctrl RNAi - SAHH RNAi	0,026	*

***Drosophila* H3K36Me2 relative quantification**

Independent samples t-tests

Sample 1 - Sample 2	p-value	significance-level
-Hcy - +Hcy	0,850	-
ctrl RNAi - SAHH RNAi	0,014	*

***Drosophila* H3K36Me3 relative quantification**

Independent samples t-tests

Sample 1 - Sample 2	p-value	significance-level
-Hcy - +Hcy	0,311	-
ctrl RNAi - SAHH RNAi	0,253	-

***Drosophila* H3K79Me1 relative quantification**

Independent samples t-tests

Sample 1 - Sample 2	p-value	significance-level
-Hcy - +Hcy	0,567	-
ctrl RNAi - SAHH RNAi	0,016	*

***Drosophila* H3K79Me2 relative quantification**

Independent samples t-tests

Sample 1 - Sample 2	p-value	significance-level
-Hcy - +Hcy	0,380	-
ctrl RNAi - SAHH RNAi	0,100	-

***Drosophila* H3K79Me3 relative quantification**

Independent samples t-tests

Sample 1 - Sample 2	p-value	significance-level
-Hcy - +Hcy	0,614	-
ctrl RNAi - SAHH RNAi	0,972	-

Supplemental Figure S1

Canton-S and *w[1118] Drosophila* larval food intake

Canton-S:

One-way ANOVA with pairwise t-tests and Games-Howell correction for multiple testing

Sample 1 - Sample 2	p-value	significance-level
-Hcy - +Hcy	0,821	-
-Hcy - +Caff.	0,048	*
+Hcy - +Caff.	0,053	-

***w[1118]* :**

Independent samples t-test

Sample 1 - Sample 2	p-value	significance-level
-Hcy - +Hcy	0,455	-

Supplemental Figure S4

***w[1118] Drosophila* larval weight**

Independent samples Mann-Whitney-U test

Sample 1 - Sample 2	p-value	significance-level
-Hcy - +Hcy	< 0,001	###

Disease Models & Mechanisms • Supplementary information

Dataset 4. Independent experimental repetitions

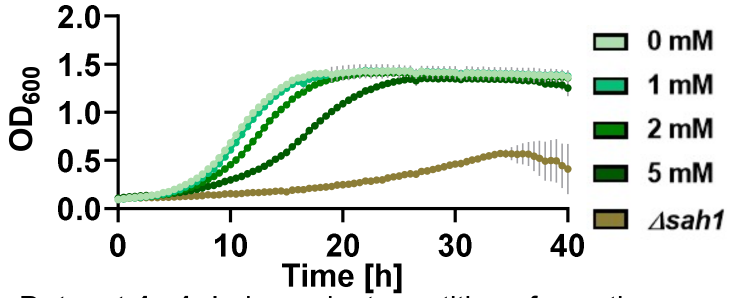

Dataset 4 - 1: Independent repetition of growth curves of wild type yeast grown in the presence or absence of Hcy supplementation as indicated, and of the yeast Δ*sah1* mutant grown without Hcy supplementation (n = 2). Repetition of Figure 2A

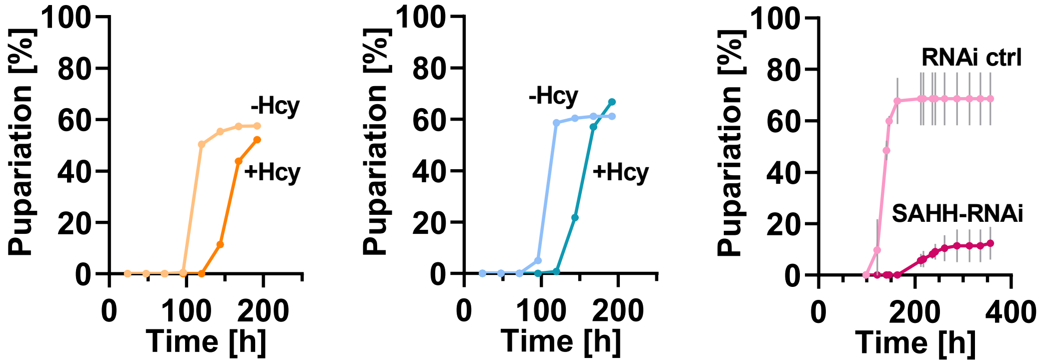

Dataset 4 - 2: Independent repetition of pupariation rates of Canton-S and *w[1118] Drosophila* larvae grown on standard fly food or food containing 20 mM Hcy (n = 1), and of *Drosophila* larvae globally expressing mCherry RNAi or SAHH RNAi grown standard food (n = 2). Repetition of Figure 2F-G & S4B

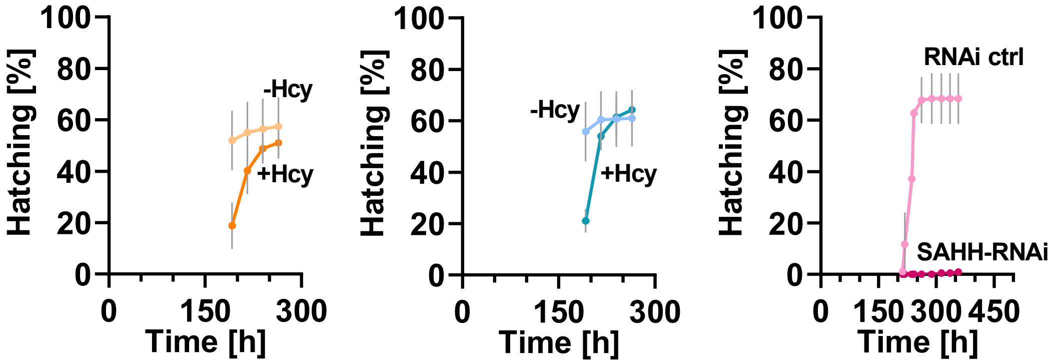

Dataset 4 - 3: Independent repetition of hatching rates of Canton-S and *w[1118] Drosophila* flies grown on standard fly food or food containing 20 mM Hcy (n = 5), and of *Drosophila* flies globally expressing mCherry RNAi or SAHH RNAi grown on standard food (n = 2). Repetition of Figure 2F-G & S4C

Disease Models & Mechanisms • Supplementary information

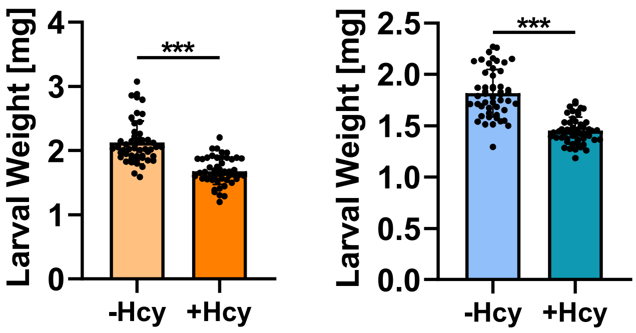

Dataset 4 - 4: Independent repetition of migratory L3 larval weight of Canton-S and *w[1118] Drosophila* larvae grown on standard fly food or food containing 20 mM Hcy (n = 50). Statistics were calculated with Students t-test or Mann-Whitney-U pairwise comparisons (* = p ≤ 0.05, ** = p ≤ 0.01, *** = p ≤ 0.001). Repetition of Figure 2E & S4A

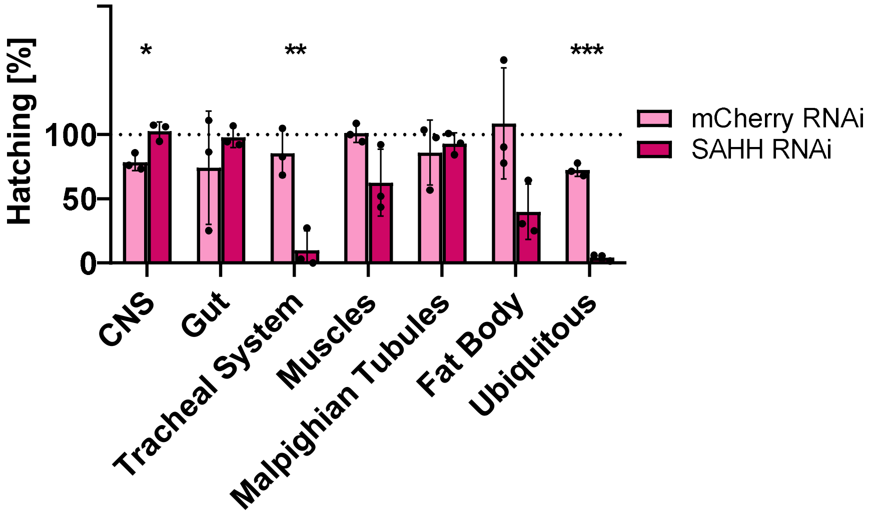

Dataset 4 - 5: Independent repetition of hatching rates of *Drosophila* flies with organ-specific expression of mCherry RNAi or SAHH RNAi grown on standard food (n = 3). Statistics were calculated with Students t-test or Mann-Whitney-U pairwise comparisons (* = p ≤ 0.05, ** = p ≤ 0.01, *** = p ≤ 0.001). Repetition of Figure 2I

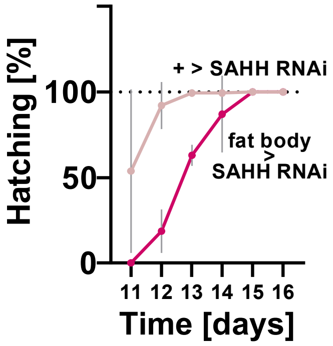

Dataset 4 - 6: Independent repetition of hatching rates of *Drosophila* progeny from the same cross with active or inactive SAHH knockdown construct in fat body grown on standard food. Of note, selective counting started on day 11 after egg deposition and given values represent total hatched flies relative to egg count (n = 3). Repetition of Figure 2J

Disease Models & Mechanisms • Supplementary information

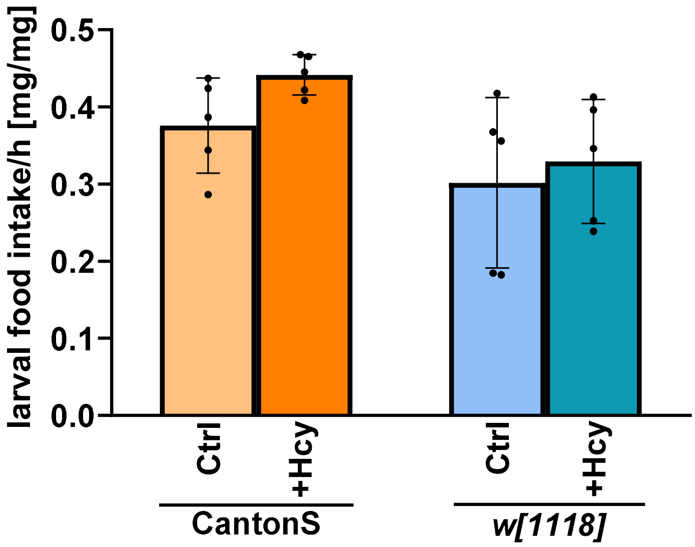

Dataset 4 - 7: Independent experimental repetition of food intake in Canton-S and *w[1118] Drosophila* larvae on standard or Hcy-supplemented food. Food intake in Canton-S and *w[1118]* non-migratory L3 larvae on standard- or Hcy-supplemented (20 mM) food per hour normalized to larval weight (n = 5). Repetition of Figure S1

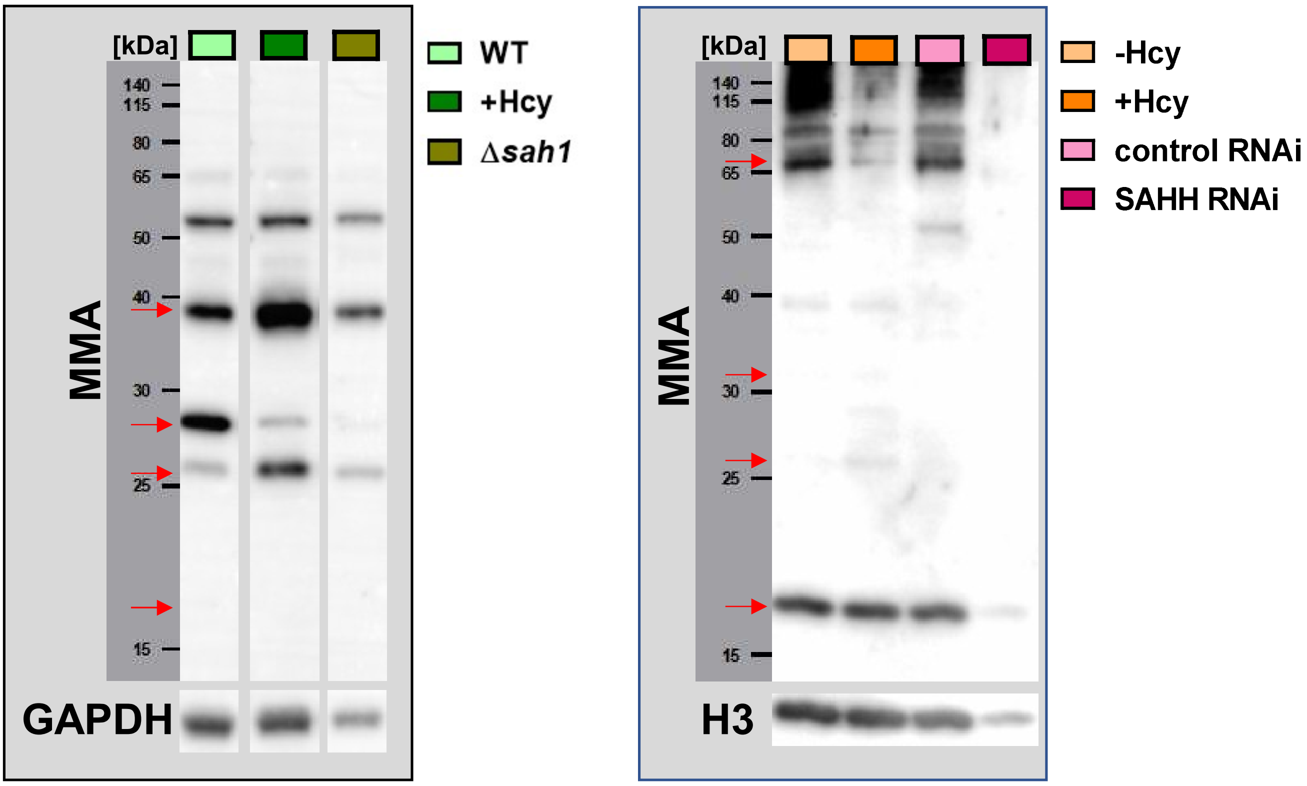

Dataset 4 - 8: Independent Repetition of western blot analysis of monomethylarginine (MMA)-containing proteins in wild type yeast grown in the presence or absence of 5 mM Hcy, and the yeast Δ*sah1* mutant grown without Hcy supplementation (left). Independent repetition of western blot analysis of MMA-containing proteins in migratory L3 *Drosophila* larvae grown on standard fly food or food containing 20 mM Hcy, and in L3 *Drosophila* larvae globally expressing mCherry RNAi or SAHH RNAi grown on standard food (right). Note, that loading volume was not adjusted for either blot, yeast western blot is a collage from one membrane with non-neighboring lines shown and for *Drosophila* western blot H3 antibody was used as loading control instead of β-actin. Repetition of Figure 4B&D

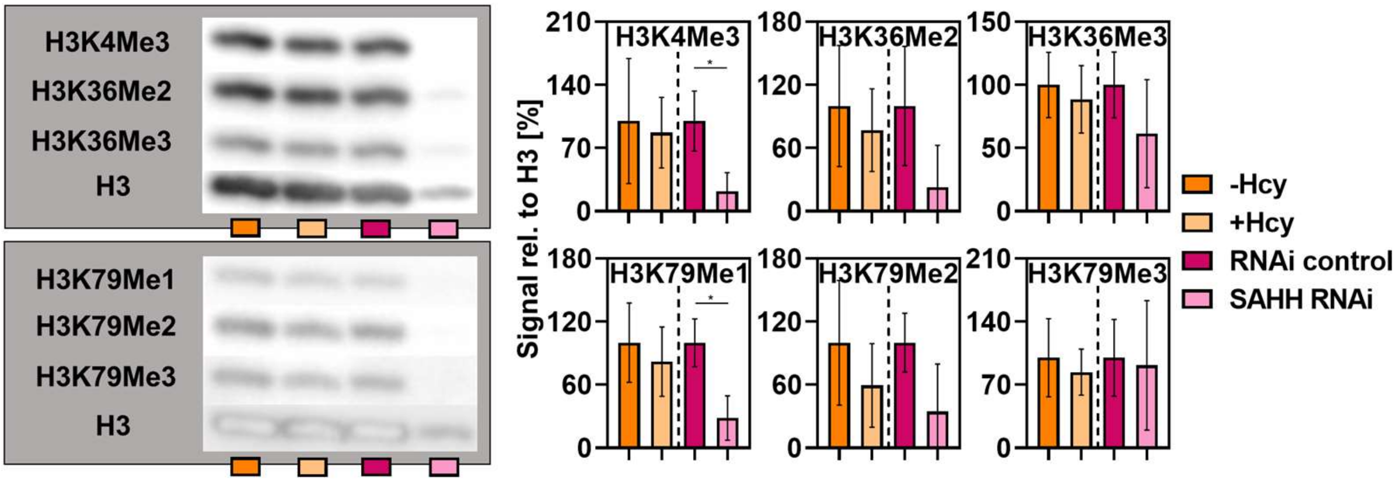

Dataset 4 - 9: Independent repetition of H3 lysine methylation in *Drosophila* dietary and genetic models. Western Blot analyses of trimethylated H3K4, di- and trimethylated H3K36, and mono-, di- and trimethylated H3K79 in migratory L3 *Drosophila* larvae grown on standard fly food or food containing 20 mM Hcy, and in L3 *Drosophila* larvae globally expressing mCherry RNAi or SAHH RNAi grown on standard food. Bar charts represent quantified signals normalized to total H3 (n = 3). Statistics were calculated with Students t-tests (* = p ≤ 0.05, ** = p ≤ 0.01, *** = p ≤ 0.001). Repetition of Figure 5B

Dataset 5. Uncropped blots

Yeast western blots – Membrane 1

Following pictures show the same blot, stripped between immuno-stainings, in order of experimental execution. Protein size standard (Pre-stained PAGEruler; Thermo Fisher Scientific) was applied to lane 1, 5 and 9. Non-supplemented wild type yeast samples were applied to lanes 2, 6, 10 and 13. Samples of wild type yeast supplemented with 5 mM Hcy were applied to lanes 3, 7, 11 and 14. Non-supplemented *Δsah1* mutant yeast samples were applied to lanes 4, 8, 12 and 15. All replicates represent independent biological samples. If applicable, red arrows mark expected band heights.

1st immuno-staining:

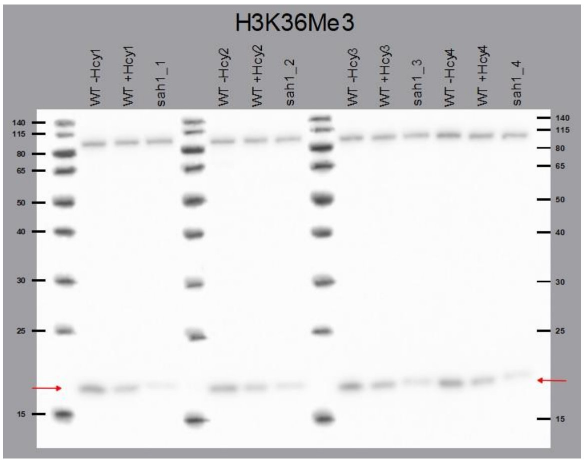

2nd immuno-staining:

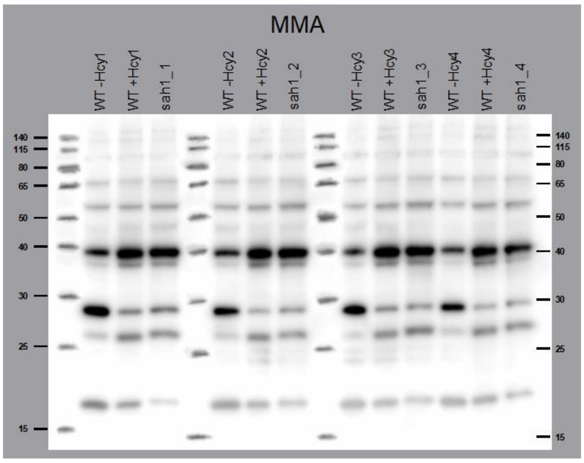

3rd immuno-staining:

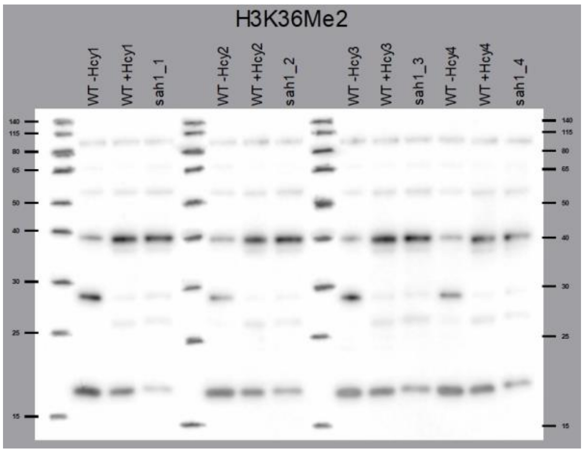

4th immuno-staining:

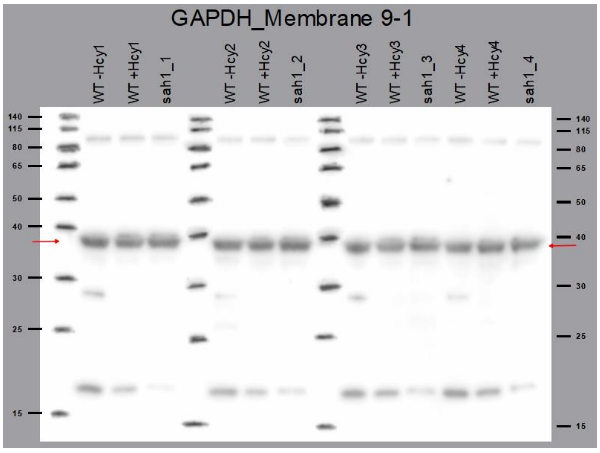

5th immuno-staining:

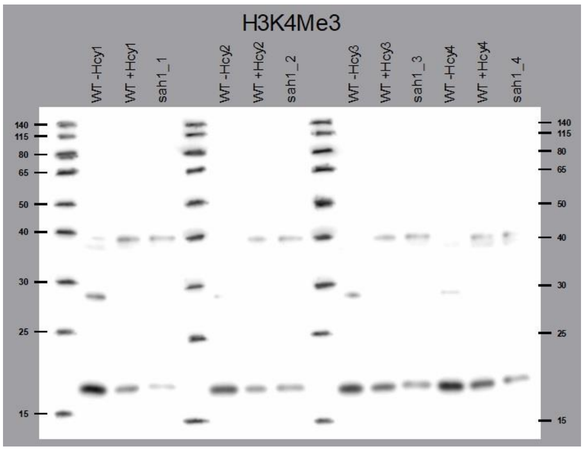

6th immuno-staining:

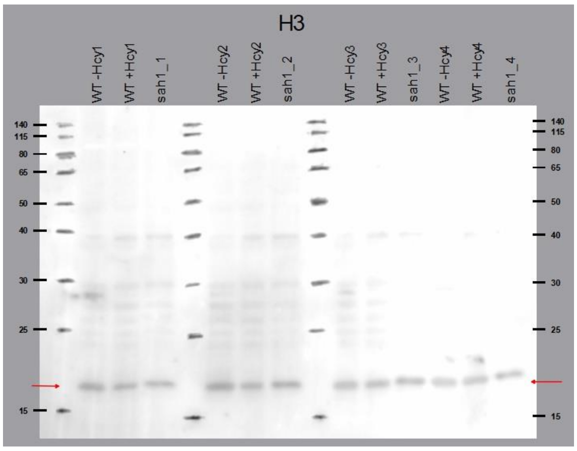

Yeast western blots – Membrane 2

Following pictures show the same blot, stripped between immuno-stainings, in order of experimental execution. Protein size standard (Pre-stained PAGEruler; Thermo Fisher Scientific) was applied to lane 1, 5 and 9. Non-supplemented wild type yeast samples were applied to lanes 2, 6, 10 and 13. Samples of wild type yeast supplemented with 5 mM Hcy were applied to lanes 3, 7, 11 and 14. Non-supplemented *Δsah1* mutant yeast samples were applied to lanes 4, 8, 12 and 15. All replicates represent independent biological samples. If applicable, red arrows mark expected band heights.

1st immuno-staining:

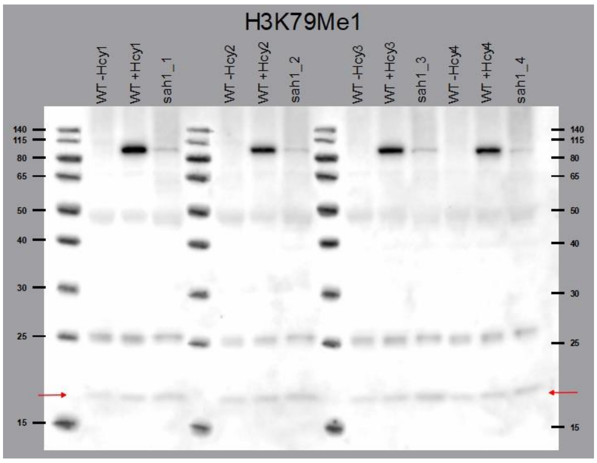

2nd immuno-staining:

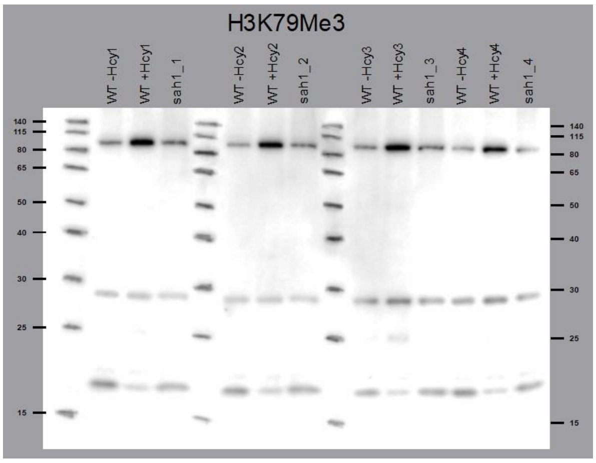

3rd immuno-staining:

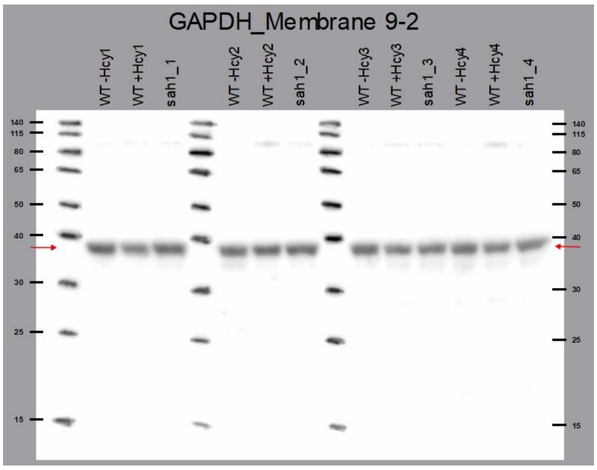

4th immuno-staining:

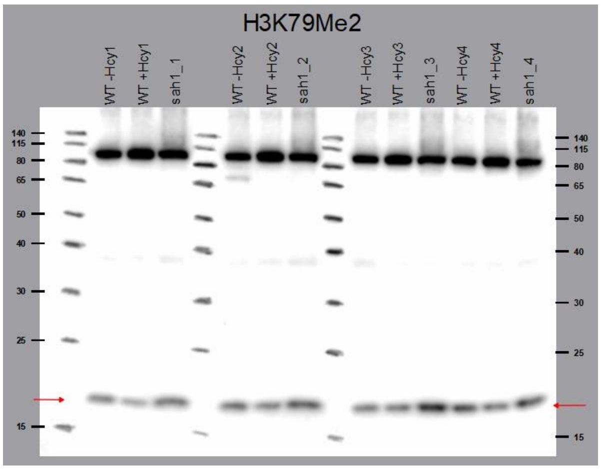

5th immuno-staining:

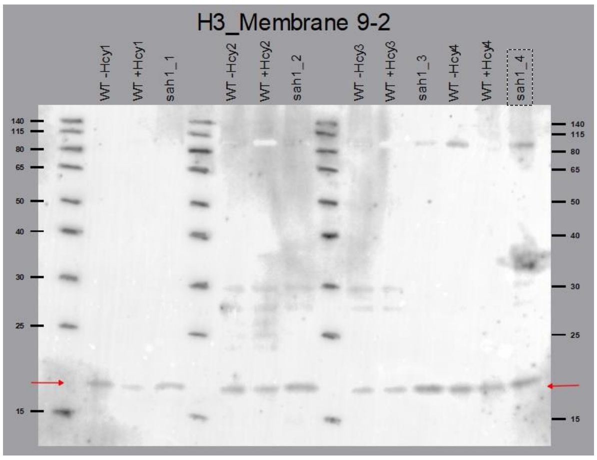

Drosophila western blots – Membrane 1

Following pictures show the same blot, stripped between immuno-stainings, in order of experimental execution. Protein size standard (Pre-stained PAGEruler; Thermo Fisher Scientific) was applied to lane 1, 6 and 11. Samples of CantonS *Drosophila* larvae grown on non-supplemented standard fly food were applied to lanes 2, 7 and 12. Samples of CantonS *Drosophila* larvae grown on 20 mM Hcy-supplemented food were applied to lanes 3, 8 and 13. Samples of Drosophila larvae globally expressing mCherry RNAi grown on non-supplemented standard fly food were applied to lanes 4, 9 and 14. Samples of Drosophila larvae globally expressing SAHH RNAi grown on non-supplemented standard fly food were applied to lanes 5, 10 and 15. All replicates represent independent biological samples. If applicable, red arrows mark expected band heights.

1st immuno-staining:

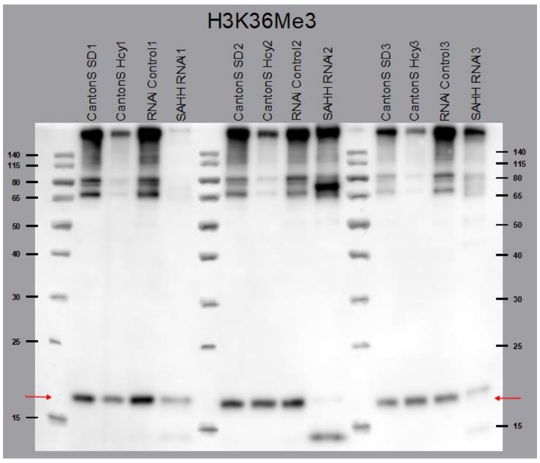

2nd immuno-staining:

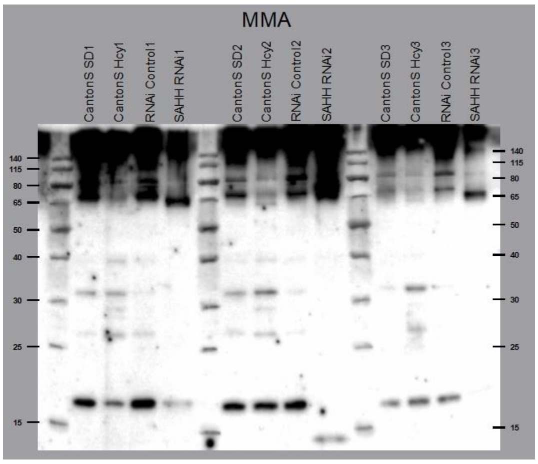

3rd immuno-staining:

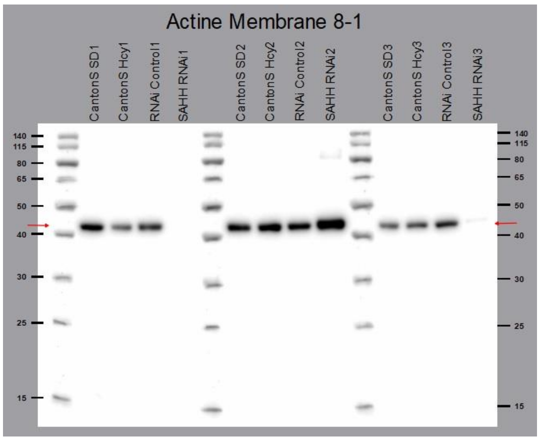

4th immuno-staining:

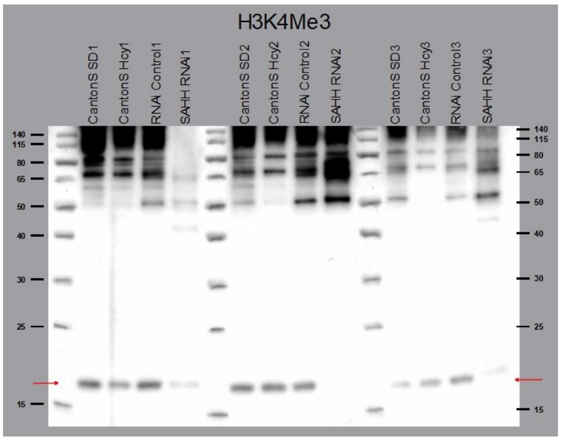

5th immuno-staining:

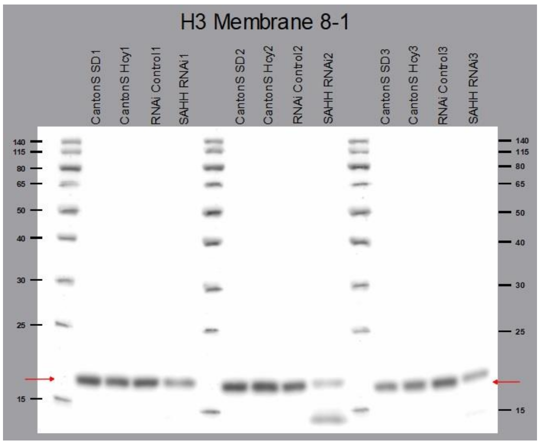

6th immuno-staining:

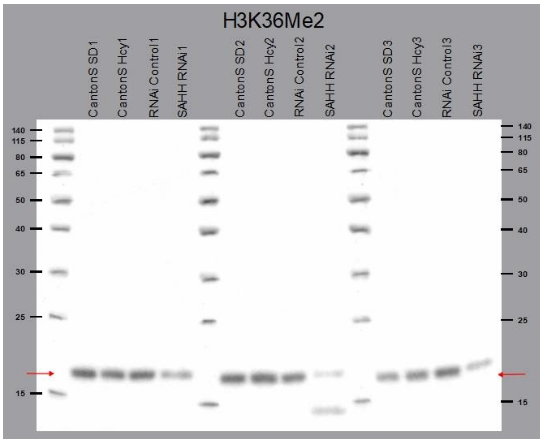

Drosophila western blots – Membrane 2

Following pictures show the same blot, stripped between immuno-stainings, in order of experimental execution. Protein size standard (Pre-stained PAGEruler; Thermo Fisher Scientific) was applied to lane 1, 6 and 11. Samples of CantonS *Drosophila* larvae grown on non-supplemented standard fly food were applied to lanes 2, 7 and 12. Samples of CantonS *Drosophila* larvae grown on 20 mM Hcy-supplemented food were applied to lanes 3, 8 and 13. Samples of Drosophila larvae globally expressing mCherry RNAi grown on non-supplemented standard fly food were applied to lanes 4, 9 and 14. Samples of Drosophila larvae globally expressing SAHH RNAi grown on non-supplemented standard fly food were applied to lanes 5, 10 and 15. All replicates represent independent biological samples. If applicable, red arrows mark expected band heights.

1st immuno-staining:

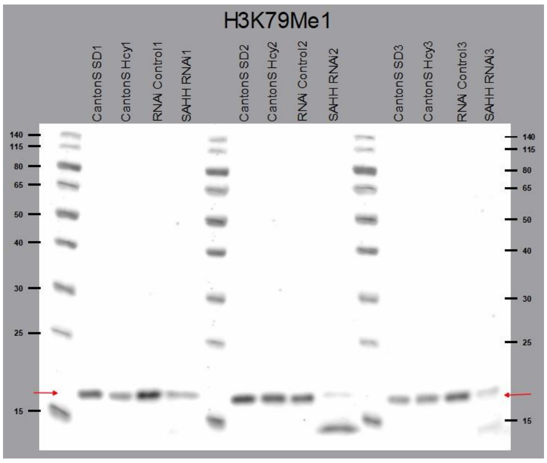

2nd immuno-staining:

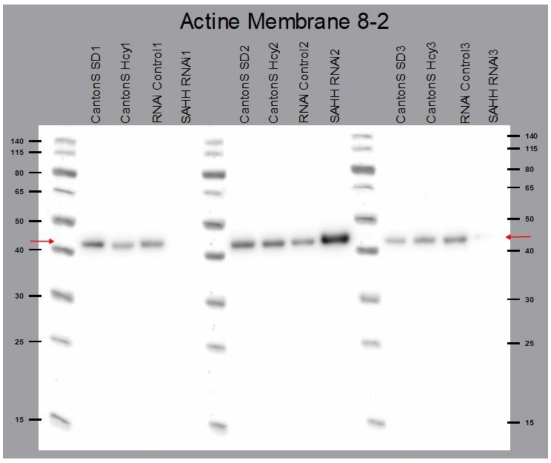

3rd immuno-staining:

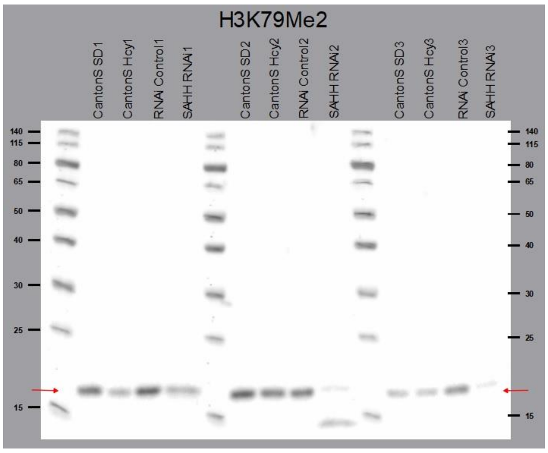

4th immuno-staining:

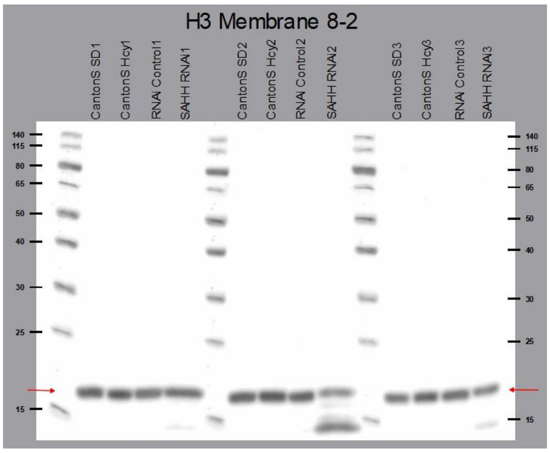

5th immuno-staining:

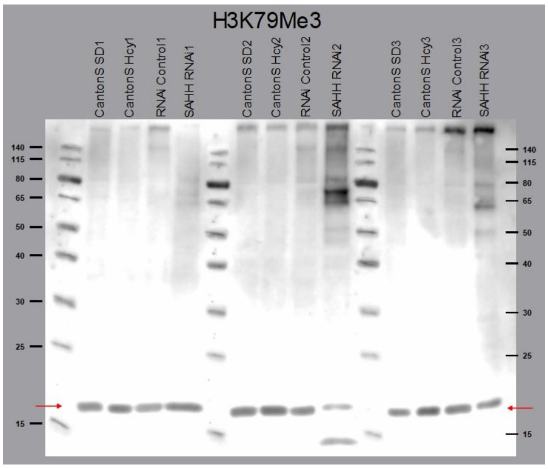

Supplement: Supplementary information [file dmm-19-052802-s1.pdf]
